# Predicting the Anti-SARS-CoV-2 Potential of Isoquinoline Alkaloids from Brazilian Siparunaceae Species Using Chemometric Tools

**DOI:** 10.3390/ijms26020633

**Published:** 2025-01-13

**Authors:** Brendo Araujo Gomes, Diégina Araújo Fernandes, Simony Carvalho Mendonça, Mariana Freire Campos, Thamirys Silva da Fonseca, Larissa Esteves Carvalho Constant, Natalia Ferreira de Sousa, Renata Priscila Barros de Menezes, Beatriz Albuquerque Custódio de Oliveira, Stephany da Silva Costa, Giovanna Barbosa Frensel, Alice Santos Rosa, Thamara Kelcya Fonseca Oliveira, Amanda Resende Tucci, Júlia Nilo Henrique Lima, Vivian Neuza Santos Ferreira, Milene Dias Miranda, Diego Allonso, Marcus Tullius Scotti, Suzana Guimarães Leitão, Gilda Guimarães Leitão

**Affiliations:** 1 Programa de Pós-Graduação em Biotecnologia Vegetal e Bioprocessos, Centro de Ciências da Saúde, Universidade Federal do Rio de Janeiro, Rio de Janeiro 21941-902, RJ, Brazil; brendoo.bc@ufrj.br (B.A.G.); ccamposmariana@ufrj.br (M.F.C.); 2 Departamento de Produtos Naturais e Alimentos, Faculdade de Farmácia, Universidade Federal do Rio de Janeiro, Rio de Janeiro 21941-902, RJ, Brazil; sy2802@ufrj.br (S.C.M.); thamirysfonseca@ufrj.br (T.S.d.F.); 3 Instituto de Pesquisas de Produtos Naturais, Universidade Federal do Rio de Janeiro, Rio de Janeiro 21941-902, RJ, Brazil; diegina@ufrj.br; 4 Programa de Pós-Graduação em Ciências Farmacêuticas, Faculdade de Farmácia, Universidade Federal do Rio de Janeiro, Rio de Janeiro 21941-902, RJ, Brazil; 5 Programa de Pós-Graduação em Ciências Biológicas, Instituto de Biofísica Carlos Chagas Filho, Universidade Federal do Rio de Janeiro, Rio de Janeiro 21941-590, RJ, Brazil; larissaestevescarvalho@gmail.com (L.E.C.C.); diegoallonso@pharma.ufrj.br (D.A.); 6 Programa de Pós-Graduação em Produtos Naturais e Sintéticos Bioativos, Universidade Federal da Paraíba, João Pessoa 58015-970, PB, Brazil; nataliafsousa@ltf.ufpb.br (N.F.d.S.); renatabarros@ltf.ufpb.br (R.P.B.d.M.); mtscotti@ccae.ufpb.br (M.T.S.); 7 Laboratório de Biotecnologia e Bioengenharia Estrutural, Centro de Ciências da Saúde, Universidade Federal do Rio de Janeiro, Bloco G, Rio de Janeiro 21941-902, RJ, Brazil; beatrizalbuquerquecp2@gmail.com (B.A.C.d.O.); stephanycosta@biof.ufrj.br (S.d.S.C.); gfrensel@peq.coppe.ufrj.br (G.B.F.); 8 Laboratório de Morfologia e Morfogênese Viral, Oswaldo Cruz Institute, Oswaldo Cruz Foundation, Rio de Janeiro 21041-250, RJ, Brazil; alicerosa@aluno.fiocruz.br (A.S.R.); thamarafonseca@ufmg.br (T.K.F.O.); artucci.bio@gmail.com (A.R.T.); julianilo@ufrj.br (J.N.H.L.); vivian.ferreira@ioc.fiocruz.br (V.N.S.F.); mmiranda@ioc.fiocruz.br (M.D.M.); 9 Programa de Pós-Graduação em Biologia Celular e Molecular, Instituto Oswaldo Cruz, Fundação Oswaldo Cruz, Rio de Janeiro 21041-250, RJ, Brazil; 10 Departamento de Biotecnologia Farmacêutica, Faculdade de Farmácia, Universidade Federal do Rio de Janeiro, Rio de Janeiro 21941-902, RJ, Brazil

**Keywords:** Brazilian folk medicine, *Siparuna* spp., SARS-CoV-2, UHPLC-MS/MS, chemometric analyses, molecular docking, dynamics simulations, ADMET predictions, bulbocapnine

## Abstract

The COVID-19 pandemic has caused over 7 million deaths globally in the past four years. *Siparuna* spp. (Siparunaceae), which is used in Brazilian folk medicine, is considered a genus with potential antiviral alternatives. This study explored the correlation between phytochemicals in *Siparuna* leaf extracts (*S. ficoides*, *S. decipiens*, *S. glycycarpa*, *S. reginae*, and *S. cymosa*) and their potential against various SARS-CoV-2 targets. In vitro assays examined interactions between the spike protein and the ACE2 receptor, protease activity, and viral replication inhibition in Calu-3 cell models. UHPLC-MS/MS analysis, processed with MZmine and evaluated chemometrically, revealed isoquinoline alkaloids with bulbocapnine, showing promising therapeutic potential. Predictions regarding absorption, distribution, metabolism, excretion, and toxicity were conducted, along with molecular docking and dynamics simulations, to evaluate protein−ligand interaction stability. The results confirmed the antiviral activity of the *Siparuna* genus against SARS-CoV-2 targets, with 92% of the extracts maintaining over 70% cellular viability at 200 μg·mL^−1^ and 80% achieving more than 50% viral activity suppression at 50 μg·mL^−1^. These findings highlight the potential of isoquinoline alkaloids as novel anti-coronavirus agents and support the need for further exploration, isolation, and testing of *Siparuna* compounds in the fight against COVID-19.

## 1. Introduction

Members of the Coronaviridae family include seasonal and highly pathogenic viruses [1]. The severe acute respiratory syndrome coronavirus 2 (SARS-CoV-2) caused a significant global impact, with over 775 million cases and 7 million deaths resulting from coronavirus disease 2019 (COVID-19) until 2024 [2,3]. The COVID-19 pandemic significantly impacted Brazil, marked by the co-circulation of multiple variants resulting from multiple independent introduction events over time. According to the WHO, Brazil reported more than 37 million confirmed cases and more than 702 thousand deaths by April 2024, making it one of the countries most affected by this disease [4,5].

SARS-CoV-2 is a positive-sense single-stranded RNA virus with a genome size of approximately 30 kb. Its genome contains several key regions that encode both structural and non-structural proteins that are essential for viral particle assembly, replication, and successful infection [6,7]. Since 2020, significant progress has been made in the research of anti-SARS-CoV-2 compounds, with different viral proteins emerging as critical targets for combating COVID-19 [1,8,9,10,11,12,13,14], such as the Spike protein, focusing on its interaction with angiotensin-converting enzyme 2 (ACE2) receptors in host cells and chymotrypsin-like cysteine protease(3CL^pro^) and papain-like protease (PL^pro^), which are primarily responsible for proteolytic cleavage of polyproteins pp1a and pp1ab during the replication process [7,15,16]. Understanding the roles of these proteins during SARS-CoV-2 infection provides valuable insights for identifying and developing effective therapeutic strategies for the management of COVID-19.

Vaccination has significantly reduced the number of severe cases and fatalities associated with COVID-19. More than 10 billion vaccine doses have been administered globally since the beginning of the pandemic, shifting the severity of the disease from a potentially fatal to a mild/moderate hazard level [3,17,18]. Despite continued vaccination efforts, including guidelines recommending vaccine boosters, SARS-CoV-2 is still circulating, and the likelihood of new strain emergence highlights the need to develop new and effective therapeutic alternatives with broad activity against different variants of SARS-CoV-2 [19,20].

Recent studies showed that plant extracts, fractions, and isolated compounds can be used to target SARS-CoV-2, either by blocking CoVs binding to host-specific receptors, such as ACE2, or preventing RNA processing by inhibiting virus-encoded proteases, such as PL^pro^ and 3CL^pro^, known as the main protease (M^pro^) [8,14,19,21,22], as well as inhibition of virus replication in cellular models (Human Lung Adenocarcinoma Cell Line Calu-3) assays [23,24].

Brazil has the greatest biodiversity in the world and a long tradition of using medicinal plants to treat major public health emergencies, making it a powerful place for discovering new antivirals [25,26,27]. Plants of the genus *Siparuna*, Siparunaceae family, are used in Brazilian folk medicine, especially in the Amazon region, for treating colds, fever, headaches, rheumatic pain, and gastrointestinal disorders, and have been studied for over 20 years by our research group [28,29,30,31].

The species *Siparuna apiosyce* (Mart.) A. DC (“limão bravo”) is mentioned in the first Brazilian Pharmacopeia due to its importance as an ingredient in syrup and cough drops [29]. Dichloromethane extracts from *Siparuna ficoides* Renner and Hausner (originally identified as *S. cristata* Poepp. and Endl. A.DC.) [32] contains the *O*-methylated flavonoids retusin and kumatakenin, which are able to inhibit the in vitro replication of SARS-CoV-2 with EC_50_ as low as 0.6 and 0.3 μM, in Calu-3 cells, respectively. Furthermore, in silico studies utilizing the Pymol, UCSF Chimera, and AutoDock Vina software v.1.1.2. programs showed a potential interaction between these flavonoids and the viral proteases 3CL^pro^ and PL^pro^, suggesting a possible mechanism of action [31,33]. Another in silico study, employing molecular docking and AutoDock Vina software v.1.1.2., analyzed the interaction of terpenes from the essential oil of *Siparuna guianensis* Aublet with SARS-CoV-2 proteins and ACE2, revealing considerable binding affinity of sesquiterpenes with the tested macromolecules, showing a promising herbal therapeutic adjuvant against SARS-CoV-2 infections [9].

Computational tools for drug design have emerged as a paramount approach for identifying bioactive compounds originating from natural products. The integration of various in vitro and in silico techniques has become a promising and advantageous strategy. These methods facilitate the precise and efficient analysis of drugs and bioactive compounds against specific protein targets [34,35]. In this manner, one can predict which metabolites are probable contributors to a specific biological activity, even prior to isolating the target compounds. This aids in the rational design of new therapeutic agents [36,37].

In this study, we combined biological and chemical screenings of *Siparuna* plant extracts with computational methods aiming to predict compounds with potential anti-SARS-CoV-2 activity from the species *Siparuna ficoides* Renner and Hausner; *Siparuna decipiens* (Tul.) A.DC., *Siparuna glycycarpa* (Ducke) Renner and Hausner, *Siparuna reginae* (Tul.) A.DC. and *Siparuna cymosa* Tolm (originally identified as *S. sarmentosa* Perkins) [32], thereby contributing to the discovery of natural product-based therapeutic options for COVID-19.

## 2. Results and Discussion

### 2.1. Siparuna spp. Extracts Against SARS-CoV-2 Targets

Five Siparunas species, *S. ficoides* (SF), *S. decipiens* (SD), *S. glycycarpa* (SG), *S. reginae* (SR), and *Siparuna cymosa* (SC), were selected based on previous literature regarding their usefulness as sources of antiviral compounds [31,33]. Thus, in this work, we tested the ability of crude ethanol extracts (CEE) from their leaves, as well as the extracts obtained after liquid-liquid extraction (hexane-HEX, dichloromethane-DCM, ethyl acetate-EtOAc, and butanol-BuOH). All the samples were subjected to different approaches in order to identify any potential inhibitory activity against SARS-CoV-2: proteases (3CL^pro^ and PL^pro^) and spike protein-ACE2 interaction.

#### 2.1.1. Siparuna Extracts Inhibit Spike Protein-ACE2 Interaction

**Figure 1:**
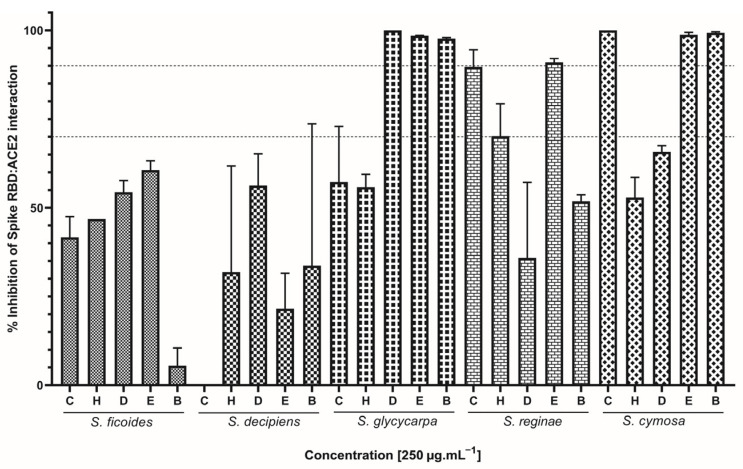
Effect of *Siparuna* extracts on spike protein-ACE2 interaction. C: crude ethanolic extract; H: hexane; D: dichloromethane; E: ethyl acetate; B: butanol. Data are expressed as means of three individual experiments (*n* = 3). Dotted lines indicate 70% and 90% inhibition.

The capacity of inhibiting RDB:ACE2 complex formation was evaluated using the immunoassay Lumit™ (Promega, Madison, WI, USA), which is a bioluminescence-based, no-wash, add-and-read kit designed for testing the potential of inhibitory samples [23]. Of the 25 assayed extracts, 16 were able to inhibit more than 50% of the Receptor Binding Domain (RBD) and ACE2 interaction (Figure 1). Notably, the addition of SG (DCM, EtOAc, and BuOH), SR (CEE and EtOAc), and SC (CEE, EtOAc, and BuOH) extracts resulted in over 90% inhibition of RBD:ACE2 complex formation. Overall, the extracts obtained from the SG, SR, and SC species proved to be the most effective in blocking the entry of SARS-CoV-2 into cells. Only SD (CEE) did not interfere with the RBD:ACE2 interaction.

#### 2.1.2. Siparuna Extracts Inhibit 3CL^pro^ Activity

**Figure 2:**
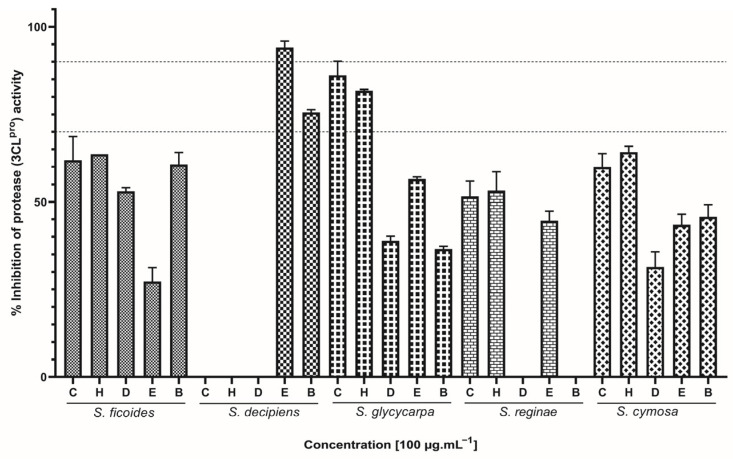
Effect of *Siparuna* extracts on 3CL^pro^ activity. C: crude ethanolic extract; H: hexane; D: dichloromethane; E: ethyl acetate; B: butanol. Data are expressed as means of three individual experiments (*n* = 3). Dotted lines indicate 70% and 90% inhibition.

SARS-CoV-2 3CL^pro^ and PL^pro^ proteolytic activities were assessed using the fluorescence resonance energy transfer (FRET) approach. This assay has already been applied to investigate potential enzyme activity inhibitors from natural sources [24]. Thirteen extracts successfully inhibited over 50% of 3CL^pro^ activity (Figure 2). Particularly, the inclusion of SD (EtOAc) extract led to over 90% inhibition of this protease in the conducted assay. Overall, SF, SG, and SC were the most promising, as all the tested extracts showed some potential for inhibiting protease activity. Only SD (CEE, HEX, and DCM) and SR (DCM and BuOH) did not interfere with 3CL^pro^ activity.

#### 2.1.3. Siparuna Extracts Inhibit PL^pro^ Activity

**Figure 3:**
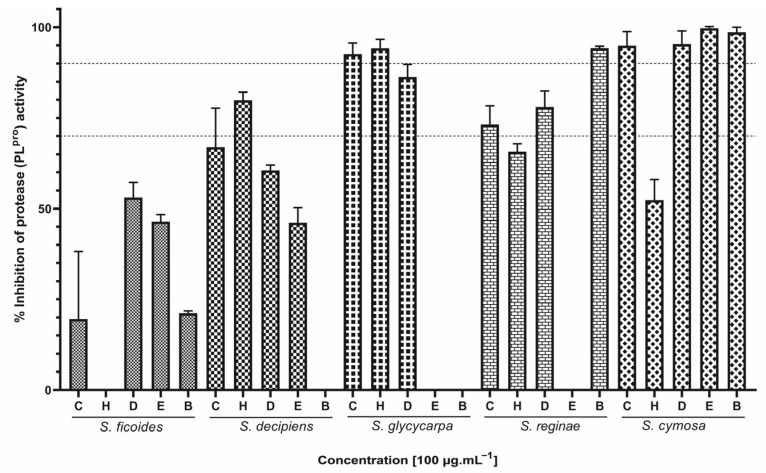
Effect of *Siparuna* extracts on PL^pro^ activity. C: crude ethanolic extract; H: hexane; D: dichloromethane; E: ethyl acetate; B: butanol. Data are expressed as means of three individual experiments (*n* = 3). Dotted lines indicate 70% and 90% inhibition.

Sixteen extracts successfully inhibited over 50% of PL^pro^ viral activity (Figure 3). Specifically, the inclusion of SG (CEE and HEX), SR (BuOH), and SC (CEE, DCM, EtOAc, and BuOH) extracts resulted in over 90% inhibition of this protease in the conducted assay. Consistent with what was observed previously for RBD:ACE2 inhibition, extracts from SG, SR, and SC demonstrated the highest inhibitory effect against PL^pro^. Only SF (HEX), SD (BuOH), SG (EtOAc and BuOH), and SR (EtOAc) did not interfere with PL^pro^ activity.

To establish a successful infection process, SARS-CoV-2 should bind to ACE2 and promote the fusion of the viral and cell membranes or induce particle endocytosis. This step is known as the virus entry process and occurs very early during infection. Natural compounds that inhibit the interaction between the S protein and ACE2 will directly aid in the management of COVID-19 by restricting the entry of SARS-CoV-2 into the host cell [38]. Similarly, inhibition of other targets, such as 3CL^pro^ and PL^pro,^ may suppress SARS-CoV-2 infection [39].

Based on our results, the species *S. cymosa* showed to be the most promising among the three evaluated activities, because all assayed extracts were able to block virus entry into the cell by inhibiting the RBD:ACE2 interaction. Additionally, they demonstrated the ability to interact with 3CL^pro^ and PL^pro^ proteases in varying proportions. The species *S. glycycarpa* also stood out for presenting one of the highest percentages of interaction against SARS-CoV-2 targets for the majority of the evaluated extracts, followed by *S. reginae*, *S. decipiens*, and *S. ficoides*, with emphasis on the extracts that showed the highest inhibition potential, namely EtOAc, BuOH, CEE, DCM, and HEX, respectively.

A previous study conducted by our research group showed that BuOH extracts from SG and SC were the most active, inhibiting 96.0 ± 1.3% and 89.5 ± 0.8% of influenza virus replication 24 h post-infection [31]. A bioassay-guided fractionation of the n-butanol extract of SG by centrifugal partition chromatography (CPC) reported annotated compounds in the CPC fractions belonging to the isoquinoline alkaloid class, flavonoid glycosides, and dihydrochalcones. Benzylisoquinoline and aporphine alkaloids were annotated in the most active fractions, whereas tetrahydroprotoberberines were the least active ones [40].

Recent studies on extracts from various plants have demonstrated their potential antiviral activities, suggesting their potential use as adjuvants in the treatment of SARS-CoV-2 infections. The ethanol extract of pomegranate peels (*Punica granatum* L.) used at different concentrations inhibited the interaction between Spike and ACE2 by up to 74%, and this effect was dose-dependent. This extract inhibited the activity of the viral 3CL^pro^ up to 80% when used at 0.2 mg⋅mL^−1^ [10]. Methanol extracts from turmeric (*Curcuma longa* L.) rhizomes, mustard (*Brassica nigra* L.) seeds, and wall rocket (*Diplotaxis erucoides* (L.) DC. subsp. *erucoides*) at 500 µg·mL^−1^ displayed significant inhibition of the 3CL^pro^ activity, resulting in inhibition protease activities of 100.0%, 90.6%, and 85.1%, respectively [11].

In vitro experiments for viral inhibition of SARS-CoV-2 revealed that whole-plant extracts (aqueous and alcoholic) of *Cissampelos pareira* L. could inhibit the virus by at least 60% SARS-CoV-2 replication in infected Vero cell cultures. The hydroalcoholic whole-plant extract showed an inhibition of 98%. The single-molecule constituents of *C. pareira* could also inhibit viral particles, with pareirarine (an isoquinoline alkaloid) showing the highest inhibition of 80%. Interestingly, the highest inhibition was shown by the whole-plant hydroalcoholic extract, which comprises various constituents. This suggests a synergistic effect of the constituents on viral inhibition [41].

The promising in vitro results obtained with plant extracts encourage the continuation of studies through their chemical analysis in the search for bioactive compounds that can be used as active principles in product formulations or as prototypes for the development of semi-synthetic derivatives, which may potentially act in the fight against emerging viral diseases, such as COVID-19 [42].

### 2.2. Chemometric Analyses Using PLS Regression Model

The analysis sought to predict the active compounds inhibiting the Spike:ACE2 interaction and the proteolytic activities of r3CL^pro^ and rPL^pro^. Twenty-five extracts from the Siparunaceae family species were subjected to LC-MS/MS (ESI and APCI) analysis in positive and negative ionization modes. The chemical data were processed and concatenated using low-level data fusion. Chemometric analyses based on binary activity values in the three target models were performed. A Partial Least Squares (PLS) analysis model was constructed to correlate LC-MS data fusion with extract inhibitory activity.

The PLS regression employed the LC-MS dataset (*m*/*z* ions and Retention Time—RT) of 75 samples as independent variables (X). The dependent variable (Y) reflected the inhibition activities against the three target models. This algorithm combines principal component analysis (PCA) and linear regression with the goal of identifying latent variables that optimize the correlation between predictors and biological activity.

To build the dataset of independent variables (X), a low-level data fusion strategy was employed. In this approach, spectral data from mass spectrometry analysis using ESI and APCI sources in positive and negative ionization modes were processed separately (normalization), concatenated, and formed an independent data matrix. The new dataset (low-level data matrix) was autoscaled. The low-level data matrix consisted of 75 rows (samples) and 460 columns (*m*/*z* ions and Retention Time), including ESI [+], 196 ions; ESI [−], 40 ions; APCI [+], 138 ions; and APCI [−], 86 ions. For the dataset of dependent variables (Y), three columns were constructed, one for each target model (Spike:ACE2; r3CL^pro^; rPL^pro^). Inhibition values were transformed into binary variables, with active samples (≥50% inhibition; 1) and non-active samples (<50% inhibition; −1).

In this study, three models, with one target focused on individual targets (Spike:ACE2, r3CL^pro^, and rPL^pro^), were created. The X-loading weight plot highlights the ions that have the most positive impact in elucidating the desired activity, specifically those exclusively present and/or more abundant in the extracts exhibiting higher activity. For these analyses, the top 10 ions that contributed the most to explaining the investigated activity were arbitrarily selected.

Upon evaluating the top 10 ions with the highest likelihood of correlation with the activity in each target, it was noticeable that among these, seven ions reappeared as those with the greatest contribution to explaining the inhibition of the Spike:ACE2 interaction, the activity of r3CL^pro^, and rPL^pro^. The presence of these seven similar ions in the three single-target models provides insights into their potential roles in the conducted experimental assays and suggests the possible multitarget activity of these compounds.

**Figure 4:**
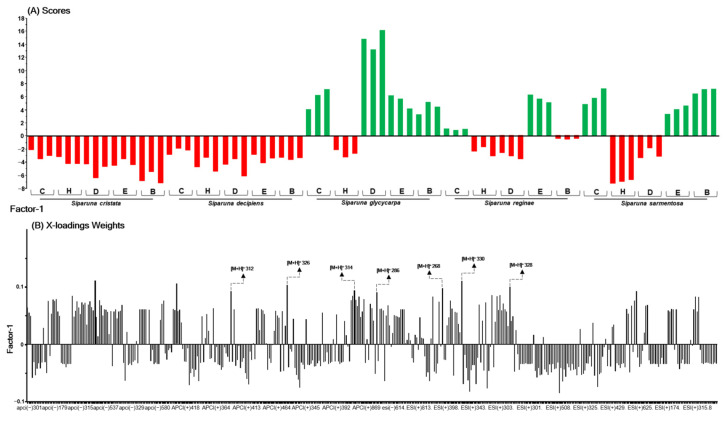
Scores (**A**), X-loadings weight (**B**) for the PLS model based on the LC-MS data obtained in the low-level data matrix (concatenated ESI and APCI [+/−]) of the 75 samples of five species of the the Siparunaceae family, and the inhibition values of the extracts on the RBD:ACE2 interaction. C: crude ethanolic extract; H: hexane; D: dichloromethane; E: ethyl acetate; B: butanol.

**Figure 5:**
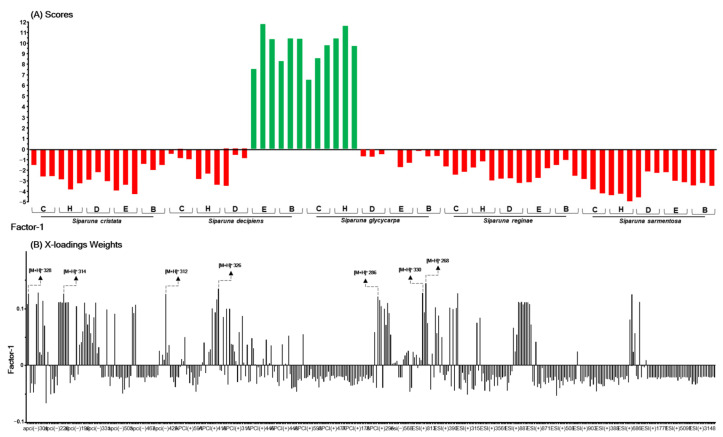
Scores (**A**), X-loadings weight (**B**) for the PLS model based on the LC-MS data obtained in the low-level data matrix (concatenated ESI and APCI [+/−]) of the 75 samples of five species of Siparunaceae family, and the inhibition values of the extracts on the r3CL^pro^ activity. C: crude ethanolic extract; H: hexane; D: dichloromethane; E: ethyl acetate; B: butanol.

**Figure 6:**
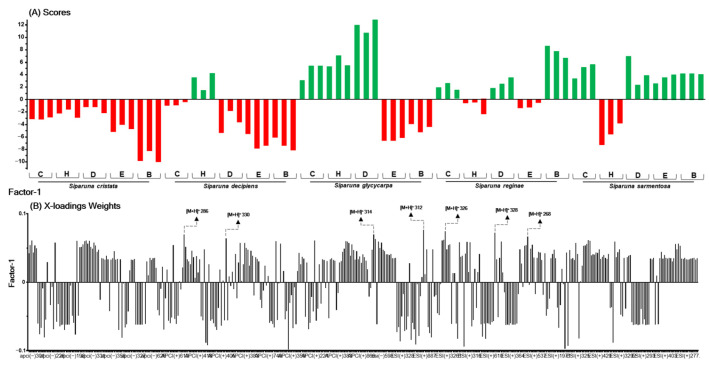
Scores (**A**), X-loadings weight (**B**) for the PLS model based on the LC-MS data obtained in the low-level data matrix (concatenated ESI and APCI [+/−]) of the 75 samples of five species of Siparunaceae family, and the inhibition values of the extracts on the rPL^pro^ activity. C: crude ethanolic extract; H: hexane; D: dichloromethane; E: ethyl acetate; B: butanol.

In Figure 4, Figure 5 and Figure 6, the green bars in the scores plot (A) represent active samples (≥50% inhibition), and red bars indicate inactive samples (<50% inhibition). The height of the bars indicates the sample’s magnitude relative to the principal component, and the direction (upward or downward) indicates the position relative to the mean. In the loadings plot (B), bar height reflects each variable’s contribution to the principal component, with higher values indicating a stronger influence on the model. Seven highlighted ions were consistently among the top 10 most relevant across the three PLS models for the Spike (RBD):ACE2, 3CL^pro^, and PL^pro^ targets, indicating their multitarget potential.

In the investigation model of Spike:ACE2 interaction inhibitors (Figure 4A,B), the explained variance was 99% (six factors), with R^2^, 0.99, and Q^2^, 0.87. The values of the Root Mean Square Error of Calibration (RMSEC) were 0.09, and those of the Root Mean Square Error of Cross-Validation (RMSECV) were 0.36. In this analysis, the ten ions with the greatest contribution to explaining the inhibition of the Spike:ACE2 interaction by the active extracts were [M + H]^+^ *m*/*z* 268, 271, 286, 312, 314, 315, 317, 326, 328, and 330.

For the model r3CL^pro^ activity inhibitors (Figure 5A,B), 99% of the variance was explained by five factors, yielding an R^2^ of 0.99 and Q^2^ of 0.92. The RMSEC value was 0.06, and the RMSECV value was 0.21. In this analysis, of the 10 ions predicted as likely significantly related to explaining the inhibition of r3CL^pro^ activity, seven ([M + H]^+^ *m*/*z* 268, 286, 312, 314, 326, 328, and 330) had already been marked for the Spike:ACE2 model. Only the ions [M + H]^+^ *m*/*z* 297, 609, and 625 are exclusive to this model.

Finally, in the model of rPL^pro^ activity inhibitors (Figure 6A,B), six factors explained 99% of the variance, resulting in an R^2^ of 0.99 and a Q^2^ of 0.88. The RMSEC value was 0.10, and the RMSECV value was 0.35. During this analysis, seven similar ions were previously predicted for the Spike:ACE2 and r3CL^pro^ models were observed again. Meanwhile, the ions exclusive to the rPL^pro^ model were [M + H]^+^ *m*/*z* 359, 397, and 603.

The values of explained variance, R^2,^ and Q^2^, as well as the lowest Root Mean Square Error (RMSE) for all obtained models, indicate a reliable fit, with the representativeness of the calibration data and the absence of overfitting. In all models, separation between the two distinct groups is evident (active and non-active samples). By selecting the 10 ions with the highest contribution, it is possible to ensure the investigation of ions with the highest likelihood of correlation with the potential activity in the three evaluated targets (multitarget), as well as streamline other computational assays, such as molecular network annotation, and studies of docking and molecular dynamics.

When evaluating the three models, it was possible to observe that the seven predicted ions (multitarget) had very close masses, within the range of [M + H]^+^ *m*/*z* 268–330. They also exhibited similar retention times (Rt: 9.0–11.6 min) and better ionization in positive mode, indicating possible nitrogenous and polar compounds. Therefore, it is conceivable that these ions belong to the same chemical class. Thus, these seven ions are of greater interest to be annotated and subjected to additional in silico assays, as they demonstrate the highest multitarget potential. Other ions predicted in the one-target models, such as *m*/*z* [M + H]^+^ 271, 315, and 317 (for Spike), 297, 609, and 625 (for 3CL^pro^), and 397, 329, and 603 (for PL^pro^), were not considered as they were not significant in the multitarget model. Additionally, the fragmentation profiles appeared to be different from those of the predicted compounds in the multitarget model, suggesting the presence of molecules of the phenolic class.

### 2.3. Annotated Compounds Using Custom Library

After data processing, a few multitarget predicted ions emerged, which had already been reported in other works by our group as anti-influenza agents [31,40]. Therefore, their APCI/ESI-MS/MS fragmentation patterns were checked (Supplementary Materials Figures S1–S7) and annotated (Table 1).

**Figure 7:**
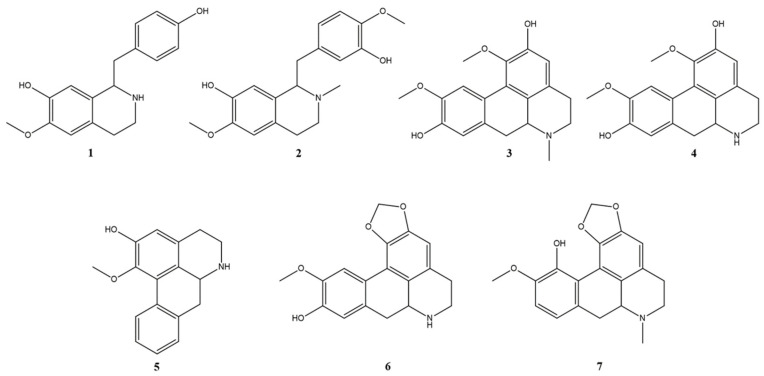
Structure of the chemical compounds identified in *Siparuna* spp. Coclaurine (**1**), reticuline (**2**), boldine (**3**), laurolitsine (**4**), asimilobine (**5**), actinodaphnine (**6**), and bulbocapnine (**7**).

These compounds present the same fragmentation profile with a first loss of CH_3_NH_2_ (−31 Da) for compounds **2**, **3**, and **7**, or NH_3_ (−17 Da) for compounds **1**, **4**, **5**, and **6**. In addition, the losses of CH_3_OH (−32 Da) followed by CO (−28 Da) were observed when an OH and an OCH_3_ group were vicinal in the aromatic ring (compounds **1**–**7**) and CH_2_O (−30 Da) followed by CO when there was a methylenedioxy group (compounds **6** and **7**) [45], characteristic of the isoquinoline alkaloids class (Figure 7).

In this way, it was possible to annotate seven isoquinoline alkaloids in the extracts that were most active against the different targets of SARS-CoV-2. Among them, five are aporphine alkaloids: actinodaphnine (**6**), boldine (**3**), laurolitsine (**4**), asimilobine (**5**), and bulbocapnine (**7**), and two are benzylisoquinolines alkaloids: coclaurine (**1**) and reticuline (**2**).

Compound **6** has been previously identified in the CEE of the *Siparuna* species investigated in the present study [31]. Compound **3** was isolated from an alkaloidal extract of *S. decipiens* [44]. Compounds **1**, **3**, **4**, and **7**, in turn, have already been identified in the BuOH extracts of *S. cymosa*, while compounds **1** and **2** have been identified in the same extract of *S. glycycarpa* [31]. Compound **5** has been isolated from *Siparuna tonduziana* Perk [43].

Natural products serve as rich sources of active compounds with diverse chemical and biochemical structures. The evaluation of antiviral activities of certain medicinal herbal extracts against CoV suggests that the phytochemicals contributing to the inhibitory effects may include flavonoids, terpenoids, and other phenolic compounds, along with alkaloids, which are prominent components in the extracts that demonstrated inhibition of viral replication and enzymatic activity [1,19,21].

Several studies have reported that flavonoids and alkaloids are the major compounds in *Siparuna* species [28,31,46]. A review of phytochemical studies of 18 species of this genus reported the isolation of 74 substances. Of these, 28 are alkaloids, 16 of which are derived from the aporphine nucleus, and liriodenine is the most cited as an isolated alkaloid among the species [30].

Alkaloids have stood out for showing significant antiviral activity and/or have been used as prototypes in the development of synthetic antiviral drugs, with several investigations of their actions against different types of coronaviruses, including SARS-CoV-2 [47,48,49]. The isoquinoline alkaloids are the second-largest class of alkaloids after indole alkaloids and may be divided into 13 classes: bis-benzylisoquinolines, aporphines, benzyltetrahydro-isoquinoline, benzylisoquinolines, benzophenanthridine, cularines, morphinans, naphythylisoquinoline, pavines, phthalideisoquinoline, promorphinans, protoberberine, and ipecac alkaloids [39].

In view of their great structural diversity, isoquinoline alkaloid derivatives obtained from different plant sources have been reported to show antiviral effects on various targets. Studies have shown that compound **6** isolated from *Actinodaphne hookeri* Meissn, could inhibit viral replication of the Herpes Simplex virus [50]; compound **3** collected from *Peumus boldus* Molina, has been investigated for the anti-influenza A virus [51]; and compound **4** isolated from the family Lauraceae, showed a significant inhibitory effect on HIV-1 integrase [52].

Kumar et al. [53] used various computational tools to identify the most eligible drug candidate with the capabilities to halt the replication of the SARS-CoV-2 virus by inhibiting M^pro^ (3CL^pro^). From a library of 100 molecules, it was observed that among the top 10 molecules, eight were alkaloid derivatives, with two aporphine alkaloids (boldine **3** and laurolitsine **4**) and laurolitsine having the highest binding affinities. This signifies that the structure of the alkaloid derivatives has the desired pharmacophoric features to perfectly bind and halt the activity of the enzyme.

The compound **7** was active against the RNA virus *Parainfluenza* (PI-3) and was employed for antiviral assessment of the compounds using Mad-ine-Darby bovine kidney and Vero cell lines [54]. An in silico study showed that compounds **1** and **2** inhibited colorectal cancer pathogenic gene products better than their native ligands, with the best binding energy [55].

Compound **1** obtained from *Cocculus hirsutus* (L.) W. Theob. was found to be promising with significant binding affinity to SARS-CoV-2 3CL^pro^ in comparison to the control and showed stable interactions with the amino acid residues present on the active site of the protease, being considered a promising anti-COVID-19 candidate [56].

Alkaloids have also been reported to have good binding affinities with important enzymes of SARS-CoV-2 in bioinformatics studies [47,57,58]. Norboldine, an aporphine alkaloid that can be extracted from the roots of *Lindera aggregate* (Sims) Kosterm., has binding potential with three target proteins: ACE2, M^pro^ (3CL^pro^), and PL^pro^ [59]. Magnoflorine is another important aporphine-type alkaloid obtained from *Tinospora cordifolia* (Willd.) Hook. f. and Thomson, and displayed a good binding energy to 3CL^pro^ and S protein of SARS-CoV-2 and ACE2 [14]. Moreover, Ellinger et al. [59] demonstrated the potential of emetine and papaverine to reduce the cytotoxic effects of SARS-CoV-2 in certain tissues.

Brown et al. [12], using a protein-based FRET-biosensor to identify inhibitors of 3CL^pro^ from SARS-CoV-2, identified 19 compounds that have EC_50_ values below 1 µM, five (N-allylnorapomorphine, R(−)-propylnorapomorphine, R(−)-2-hydroxyapomorphine, and R(−)-2,10,11-trihydroxy-N-propylnoraporphine) of which were aporphine alkaloids not previously identified as 3CL^pro^ inhibitors, suggesting a promising starting point for structure-activity-relationship studies to develop new antiviral compounds.

In exploring the potential structure-activity relationships of isoquinoline alkaloids, Sharma et al. [39] underscored the significance of a nitrogen atom, particularly quaternary, and the presence of methyl groups within the structure of bioactive molecules. Compounds with such attributes, such as berberine, offer indirect support in combating diseases, enhancing immune responses (immunomodulation), or modulating pro-inflammatory reactions [60]. Moreover, other methoxylated alkaloids, including tetrandrine, neferine, berbamine, cephranthine, and hernandezine, have demonstrated substantial potential for combating SARS-CoV-2 infection by impeding viral entry into host cells. These compounds disrupt various pathways involved in the entry stage, including the inhibition of clathrin-mediated fusion induced by Ca^2+^ influx, as well as the blockade of two-pore channel 2 and TRPMLs [61,62,63,64]. Furthermore, these compounds may target other crucial phases of viral infection, such as replication. Ghareeb et al. [65] and Varghese et al. [66] demonstrated that berberine inhibits 3CL^pro^ and PL^pro^ activity while also suppressing the expression of both the E-gene and RNA-dependent RNA polymerase (RdRp). These findings collectively highlight the multifaceted potential of isoquinoline alkaloids in combating viral infections and underscore their importance in therapeutic development.

### 2.4. Siparunaceae Extracts Inhibit SARS-CoV-2 Replication in Human Infected Lung Cell Model

**Figure 8:**
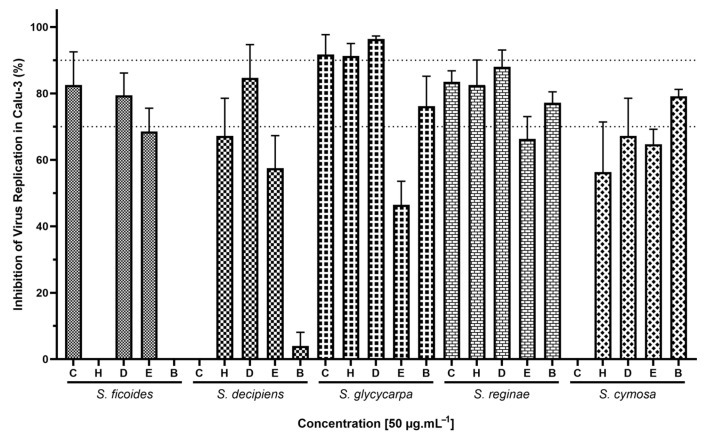
Calu-3 cells infected with SARS-CoV-2 at an MOI of 0.01, were treated with 25 extracts from *Siparuna* species at 50 μg·mL^−1^ for 24 h at 37 °C, 5% CO_2_. C: crude ethanolic extract; H: hexane; D: dichloromethane; E: ethyl acetate; B: butanol. Viral titers were assessed by plaque assay in PFU/mL and compared to infected and untreated controls (*n* = 4). Dotted lines indicate 70% and 90% inhibition.

After confirming that the extracts exhibited inhibitory activity against viral proteases and the interaction between the viral spike protein and cellular ACE2, we assessed their ability to inhibit viral replication in a complex cellular system. For this purpose, experimental assays were performed using the human pneumocyte line Calu-3, acknowledged as an optimal model for in vitro infection and the screening of anti-coronavirus agents due to its elevated susceptibility to SARS-CoV-2 via a transmembrane serine protease 2 (TMPRSS2)-dependent entry mechanism [53,67,68,69]. Initially, Calu-3 cells were subjected to infection at a multiplicity of infection (MOI) of 0.01. The extracts were then tested for their antiviral efficacy at a concentration of 50 μg·mL^−1^ over a 24-h treatment period to identify the plant species demonstrating the most effective antiviral properties (Figure 8). The extracts’ impact on viral replication in Calu-3 cells revealed that 20 (80%) extracts resulted in a reduction of viral activity by over 50%, and 10 (40%) of them inhibited more than 80% of viral replication under the same previously described conditions.

**Figure 9:**
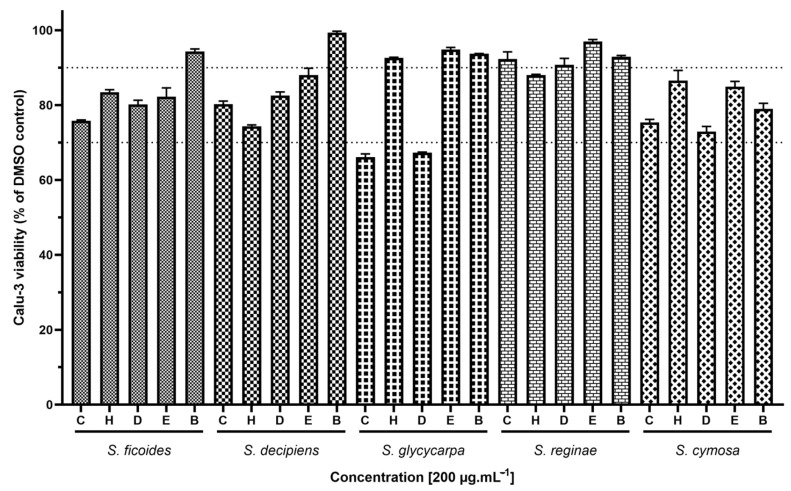
Calu-3 cells were treated with the 25 extracts of *Siparuna* species at 200 μg·mL^−1^ for 72 h at 37 °C and 5% CO_2_. C: crude ethanolic extract; H: hexane; D: dichloromethane; E: ethyl acetate; B: butanol. Cell viability was assessed by analyzing the activity of the enzyme lactate dehydrogenase (LDH) and comparing it with that of the untreated controls. (*n* = 3). Dotted lines indicate 70% and 90% of inhibition.

To evaluate the toxicity of compounds in the Calu-3 cell model, we exposed the cells to the maximum possible concentration of the extract, ensuring that the dimethyl sulfoxide (DMSO) content did not exceed 1% in the culture medium to prevent interference with cellular viability from DMSO (Figure 9). It was observed that the components of the extracts were non-toxic to this cellular model, as 23 (92%) extracts facilitated at least 70% cellular viability, with 18 (72%) achieving viability above 80%.

These results indicate that the concentration required for the extracts to cause any cellular damage is at least four times higher than the concentration needed to inhibit at least 50% of viral replication. This significant difference between the therapeutic efficacy and cytotoxicity underscores the potential safety and effectiveness of Siparunaceae extracts as anti-SARS-CoV-2 agents. The ability to achieve substantial antiviral effects at low concentrations, where cellular damage is minimal, highlights their promise in the bioprospecting of new anti-SARS-CoV-2 compounds. These results not only reinforce previous observations regarding the potent antiviral capabilities of Siparunaceae extracts, as discussed by our research group and others [31,33,70], but also highlight the specific impact of these botanical compounds on a relevant model of respiratory viral infection. The high efficacy of certain extracts in blocking SARS-CoV-2 replication suggests that bioactive compounds within these plants, possibly alkaloids and flavonoids, interact with viral particles or cellular mechanisms in a manner that markedly disrupts the viral life cycle.

This potent antiviral effect, particularly in a respiratory cell model, supports the potential therapeutic application of Siparunaceae extracts in treating infections caused by SARS-CoV-2. Further pharmacological exploration is required to identify the active constituents and to understand their mechanisms of action. Such research could pave the way for the development of novel antiviral agents derived from Siparunaceae, offering a natural and effective option for managing pandemics similar to COVID-19.

### 2.5. Results from ADMET Predictions

Initially, absorption, distribution, metabolism, excretion, and toxicity (ADMET) predictions were conducted using the Swiss ADME and Osiris DataWarrior platforms (Supplementary Materials Table S1). The aim of this analysis was to identify compounds with favorable pharmacokinetic, pharmacodynamic, and pharmacological profiles, indicating their potential for further development. All analyzed compounds have a high probability of being absorbed through the gastrointestinal tract and crossing the blood-brain barrier (BBB). Additionally, none of them showed a significant probability of toxicity to the reproductive system, nor were they likely to be skin irritants, mutagenic, or tumorigenic. The only exception was compound **1**, which showed a high probability of tumorigenic activity.

The potential interactions of the seven identified compounds with the five major isoforms of cytochrome P450 (CYP1A2, CYP2C19, CYP2C9, CYP2D6, and CYP3A4) were investigated (Table 2). Interestingly, only the compound bulbocapnine was predicted to have the potential to inhibit all CYP isoforms, especially the CYP2C19 and CYP2C9 isoforms, for which no other compound was predicted as an inhibitor.

**Figure 10:**
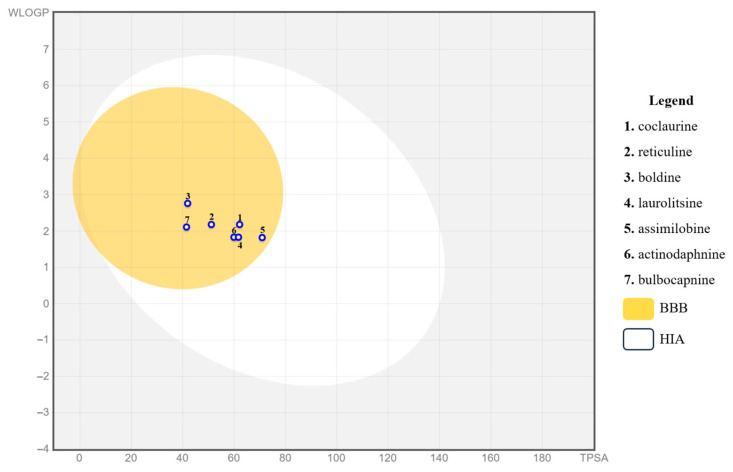
Boiled egg diagram of the seven compounds under study. Points located in the yellow area indicate molecules predicted to passively permeate the blood−brain barrier (BBB). The white area represents the molecules passively absorbed by the human gastrointestinal tract (HIA). Blue points indicate molecules predicted to be effluxed from the central nervous system by P-glycoprotein.

The gastrointestinal absorption and brain penetration of the seven identified compounds were illustrated using the Boiled Egg diagram, as depicted in Figure 10, using the Swiss ADME web tool. In the figure, the white region represents the area with the highest likelihood of absorption by the human gastrointestinal system, while the yellow portion (the yolk) indicates the region with the highest likelihood of brain penetration. The diagram revealed that all examined compounds fell within the yellow region, suggesting a high probability of absorption by the human gastrointestinal tract and brain permeation.

### 2.6. Docking Analysis

The seven compounds under study were subjected to molecular docking simulations to analyze the probability of interaction with the proteases of SARS-CoV-2, with the results demonstrated in three different scoring functions corresponding to the MolDock Score, Rerank Score, and PLANTS Score (the binding energy values of the compounds under study for the proteins related to the inhibition of the SARS-CoV-2 virus can be viewed in Supplementary Materials Tables S2–S7).

Prior to conducting the docking, redocking was performed to validate the methodology under study, given that redocking corresponds to the Root Mean Square Deviation (RMSD) value of the ligand. It is considered ideal for the RMSD value in docking to be equal to or less than 2.0 Å. Thus, an RMSD less than 2.0 Å is widely accepted as a discriminant in reproducing a known binding mode, indicating the success of the method [71]. The ligands were selected based on their similarity to the compounds under study according to the criteria of heavy atoms, chemical groups, and molecular mass. The co-crystallized ligands available in the crystallography made available in the PDB are representative and biologically relevant: since they were experimentally validated, it has been demonstrated in reference articles [72,73,74,75,76,77] that they represent a relevant biological interaction with the target studied. In this way, it is possible to show that the docking protocol is capable of predicting a pose similar to the experimental one. According to the obtained RMSD value, it can be observed that it was below 2.0 Å (Table 3). These data indicated that the generated pose correctly positioned the ligand in the active site, suggesting that the program provided values that were considered satisfactory for docking validation.

Regarding the 3D structures (PDB 3SCI and 6M0J) of the Spike protein [72,73], it was observed that they do not have co-crystallized ligands. The active site of these proteins was identified through the reference article of the PDB and the use of molecular pocket predictions such as the Bite Net Platform—Skoltech I Molecule, 2022 [78]. The coordinates used for the identification of the active site of the Spike protein were X: 12.70 and −32.26, Y: −6.44 and 21.10, and Z: 60.95 and −8.16 for PDBs 3SCI and 6M0J, respectively. For the Spike protein, remdesivir was used as a positive control [79].

The docking results regarding the total probability values for the enzymes under study can be viewed in the main text and in the Supplementary Materials (Supplementary Materials Tables S2–S7). From this perspective, it can be observed that the compounds under study exhibited negative binding energy values in all enzymes studied, indicating interactions with the targets under study. The following sections discuss the results related to the compounds’ affinity to the targets under study.

#### Docking in Different Targets of SARS-CoV-2

The results of the probability of activity for the target Spike protein, 3CL^pro^, and PL^pro^ are shown in Table 4. All test compounds displayed *p* enzyme values above 0.6. In the Spike protein, the PDB ligand (positive control) exhibited the highest affinity (*p* enzyme 0.98). Notably, bulbocapnine (**7**) had the second-highest activity probability for the Spike protein in PDB 3SCI (*p* = 0.77), while reticuline (**2**) ranked second for PDB 6M0J (*p* = 0.78). These compounds maintained high affinity for the *p* enzyme, with compound **7** at *p* = 0.73 and compound **2** at *p* = 0.77. For 3CL^pro^, compound **2** had the highest *p* enzyme value (*p* = 0.90), followed by boldine (**3**) (*p* = 0.89). The *p* enzyme value of the PDB was lower (*p* = 0.72) than that of these compounds. Compound **7** also had the highest total probability value for the 3D structure with code 6M2N (*p* = 0.94), and for PDB 7B3E, compound **3** had the highest activity probability value (*p* = 0.89). In turn, for PL^pro^, the PDB ligand showed the highest affinity, with the *p* enzyme equal to 0.99. Actinodaphine (**6**) stood out with a probability value of *p* = 0.62 for PDB 7LBR, while for PDB 7TZJ, the highest value (*p* = 0.84) was from compound **7**. Considering both targets, compounds **6** (*p* Enzyme 0.69) and **7** (*p* Enzyme 0.66) had the highest probability values after the ligand.

**Figure 11:**
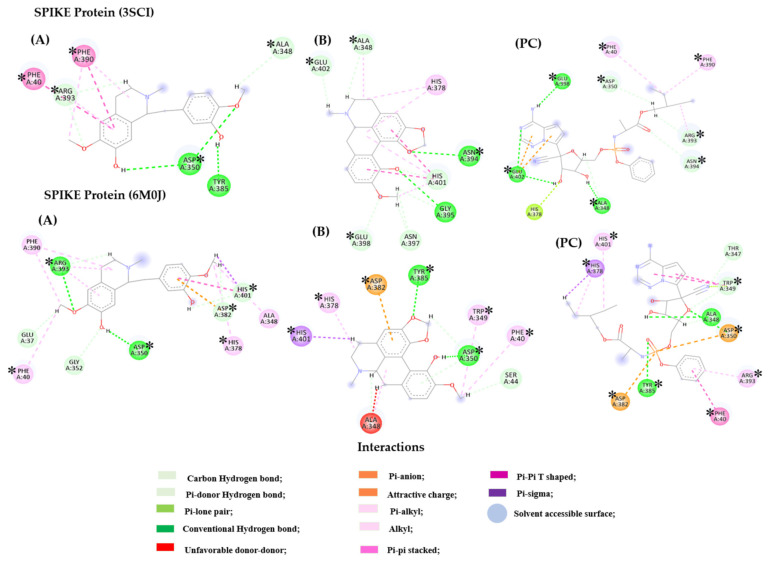
2D and 3D interactions among compound reticuline (**2**) (**A**), bulbocapnine (**7**) (**B**), positive control (**PC**), and targets of SARS-CoV-2 (PDBs 3SCI and 6M0J—Spike). Residues: Ala (Alanine), Arg (Arginine), Asn (Asparagine), Asp (Aspartic acid), Glu (Glutamic acid), Gly (Glycine), His (Histidine), Phe (Phenylalanine), and Tyr (Tyrosine). * Indicates the occurrence of similar residues in the interactions between compounds and PC.

In the analysis of the Spike protein (PDB 6M0J), compound **2** demonstrated polar interactions (hydrogen bonding), including hydroxyl groups (OH) and oxygen atoms of methoxyl groups (OCH_3_) with the residues Ala 348, Arg 393, Asp 350, Asp 382, Glu 37, Gly 352, His 378, His 401, Phe 40, and Phe 390. Moreover, hydrophobic interactions were observed, including Pi-alkyl, alkyl, Pi-pi stacked, and Pi-pi T interactions, through residues Arg 393, Asp 382, His 401, and Phe 390. In PDB 3SCI, compound **7** showed hydrogen interactions with residues Ala 348, Asn 394, Asn 397, His 401, Gly 395, Glu 398, and Glu 402, accompanied by hydrophobic interactions with Ala 348, His 378, and His 401. The similar residues between the compounds and the positive control (PC) are in bold. The molecular interactions of compounds **2** and **7**, along with the positive control (PDB ligand), with the Spike protein (PDBs 3SCI and 6M0J) are illustrated in Figure 11.

For the Spike protein, the highest affinity was observed for the positive control remdesivir, corresponding to *p* Enzyme equal 0.98. This compound is still used worldwide as a medication for the treatment of COVID-19. It prevents the entry and replication of the virus in the body, thereby reducing the infection process [80,81,82]. Thus, it emerges as an excellent alternative as a positive control for this target. It was also noted that remdesivir exhibited the highest probability values for the PDB structures 3SCI (*p* = 1) and 6M0J (*p* = 0.97). Interactions with the Spike protein, like those demonstrated by remdesivir, were observed for compounds **5** and **7**, corresponding to all residues for polar and non-polar interactions, except His 378, in the structure PDB 3SCI. Meanwhile, for the PDB 6M0J structure, there were similar interactions with the positive control in all residues except for Ala 348 and Thr 347 (polar interactions) and Asp 350 (non-polar interactions). Due to the similarity of interactions between the standard medication against COVID-19, remdesivir, and compounds **2** and **7**, one could speculate a possible similar inhibitory action on the specified target, the Spike protein.

**Figure 12:**
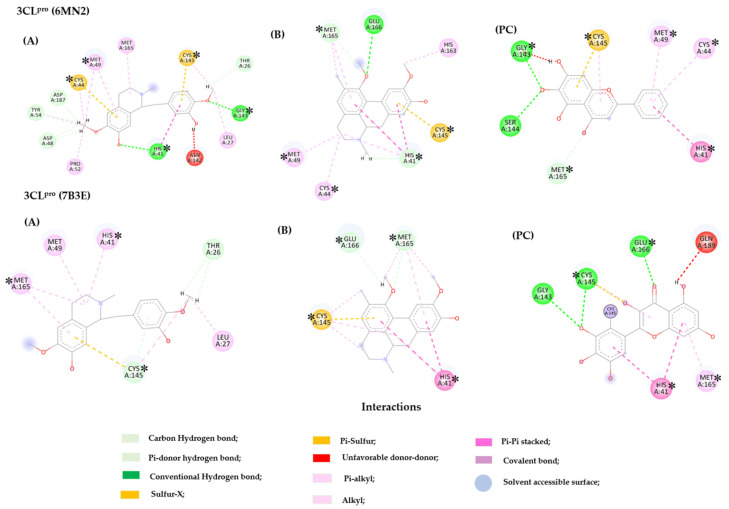
2D and 3D interactions among compound reticuline (**2**) (**A**), boldine (**3**) (**B**), positive control (**PC**), and targets of SARS-CoV-2 (PDBs 6MN2 and 7B3E—3CL^pro^). Residues: Ala (Alanine), Arg (Arginine), Asn (Asparagine), Asp (Aspartic acid), Glu (Glutamic acid), Gly (Glycine), His (Histidine), Phe (Phenylalanine), and Tyr (Tyrosine). * Indicates the occurrence of similar residues in the interactions with the compounds and PC.

In the 3CL^pro^ enzyme (PDB 6M2N), compound **2** involved polar interactions through the -OH and -OCH_3_ groups with residues Asp 48, Asp 187, Asn 142, Cys 44, Cys 145, Gly 143, His 41, Leu 27, Met 49, Pro 52, and Tyr 54. Pi-sulfur interactions were observed with aromatic rings, along with alkyl and Pi-alkyl interactions involving residues Cys 44, Cys 145, His 41, Met 49, and Met 165. For PDB 7B3E, compound **3** hydrogen bonding interactions were observed with residues Glu 166 and Met 165, accompanied by prominent hydrophobic interactions with Cys 145, His 41, and Met 165. Similar residues between the compounds and PC are in bold. These interactions demonstrated that the studied compounds exhibited important interactions with both the 3D structures under investigation (Figure 12).

The tested compounds (**2** and **3**) formed important interactions with residues constituting the enzyme’s active site, including Cys 145 and His 41, which are part of the enzyme’s catalytic dyad. The hydrogen bond interaction established by residue Glu 166 is crucial in the enzyme’s main chain. Hydrophobic interactions formed by residues Cys 44, His 41, and Met 49 are important in the S2 subsite. The hydrophobic interaction established by residue Met 165 is crucial for maintaining the enzyme’s intermediate chain.

**Figure 13:**
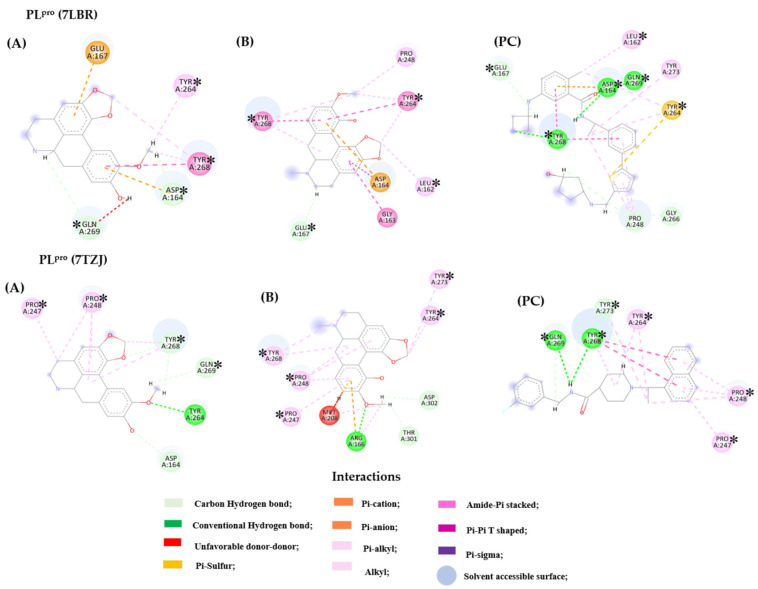
2D and 3D interactions between compound actinodaphnine (**6**) (**A**), bulbocapnine (**7**) (**B**), positive control (**PC**), and targets of SARS-CoV-2 (PDBs 7LBR and 7TZJ). Residues: Ala (Alanine), Arg (Arginine), Asn (Asparagine), Asp (Aspartic acid), Glu (Glutamic acid), Gly (Glycine), His (Histidine), Phe (Phenylalanine), and Tyr (Tyrosine). * Indicates the occurrence of similar residues in the interactions with the compounds and PC.

In the PL^pro^ enzyme structure (PDB 7LBR), compound **6** formed hydrogen bonds with residues Asp 164, Gln 269, Tyr 264, and Tyr 268 and hydrophobic interactions with Asp 164, Tyr 268, and Glu 167, interacting with these residues in the flexible β-hairpin loop BL2 of the enzyme. In this region of greater importance (β-hairpin loop BL2), the most relevant residues present in the PDB 7TZJ structure formed polar (Arg 166, Asp 302, and Thr 301) and non-polar (Arg 166, Met 208, Pro 247/248, and Tyr 264/268/273) strongly interacted with compound **7**. Overall, the studied compounds **1** and **5** exhibited significant interactions with the active sites of the enzymes, demonstrating potential therapeutic relevance. In bold are the similar residues between the compounds and PC. Figure 13 shows the interactions of compounds **6**, **7**, and the PDB ligand (PC) with PL^pro^ (PDBs 7LBR and 7TZJ).

When evaluating all compounds across the three targets, it can be observed that actinodaphnine (**6**), boldine (**3**), bulbocapnine (**7**), and reticuline (**2**) formed interactions of greater relevance with the Spike protein, as well as with the active sites of the 3CL^pro^ and PL^pro^ enzymes. However, it is worth noting that compounds **2** and **7** exhibited interaction probabilities in the active site of more proteins, with significant *p* enzyme values. Thus, aiming for expediency in the search for compounds with potential activity, further in silico assays (Molecular Dynamic Analysis) were conducted, focusing solely on these two compounds.

### 2.7. Molecular Dynamics Analysis

The investigation of potential drugs and their interactions with target proteins is a crucial step in the development of effective therapies, especially in vital areas such as pharmacology. The molecules bulbocapnine (**7**) and reticuline (**2**) have emerged as the most promising molecules in molecular docking analysis, standing out for their ability to interact with all the proteins analyzed. This characteristic makes them particularly intriguing for further investigations, such as molecular dynamics studies.

Molecular dynamics play a fundamental role in evaluating the stability of interactions between molecules and protein active sites. For this investigation, only one specific PDB file was selected for each protein of interest. For example, PDB file 6M2N was chosen for the 3CL^pro^ protein, while 7TZJ was selected for the PL^pro^ protein, and file 6M0J for the Spike protein.

**Figure 14:**
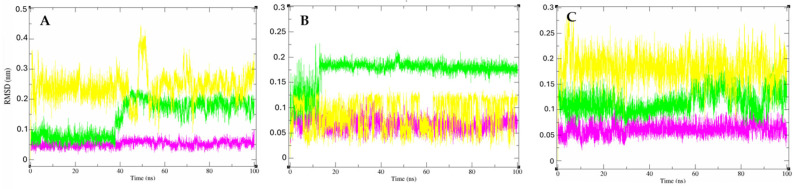
Root mean square deviation (RMSD). (**A**) Spike (6M0J), (**B**) 3CLpro (6M2N), and (**C**) PL^pro^ (7TZJ). bulbocapnine (**7**) (Pink), reticuline (**2**) (green), and inhibitor protein (yellow).

Initially, root mean square deviation (RMSD) analyses were conducted to assess the structural stability of the receptor structure over time. These analyses provided valuable insights into the stability of the interactions of the studied molecules. Notably, it was observed that compound **7** demonstrated greater interaction stability with target proteins compared to compound **2**. Furthermore, compound **7** also exhibited greater interaction stability than the standard inhibitor ligands for each protein. This finding is crucial, indicating that the molecular interactions between compound **7** and the enzymes are relatively more stable and long-lasting, which are key factors in the development of effective drugs. These findings are shown in Figure 14.

**Figure 15:**
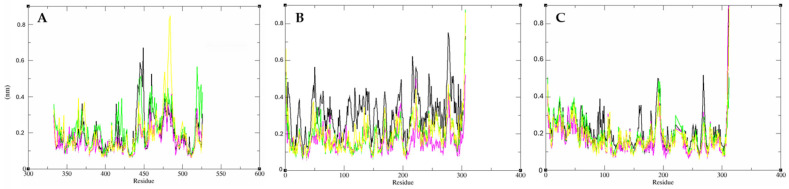
Root mean square fluctuation (RMSF). (**A**) Spike (6M0J), (**B**) 3CL^pro^ (6M2N), and (**C**) PL^pro^ (7TZJ). Complex protein bulbocapnine (**7**) (pink), complex protein reticuline (**2**) (green), complex protein inhibitor protein (yellow), and protein alone (black).

Subsequently, residue flexibility was analyzed using root mean square fluctuation (RMSF) values, revealing similar patterns between the isolated protein and the protein−ligand complex throughout the simulation. This consistency indicates the stability of the molecule-protein interaction, with no significant variations in the protein that could hinder the interaction (Figure 15).

**Figure 16:**
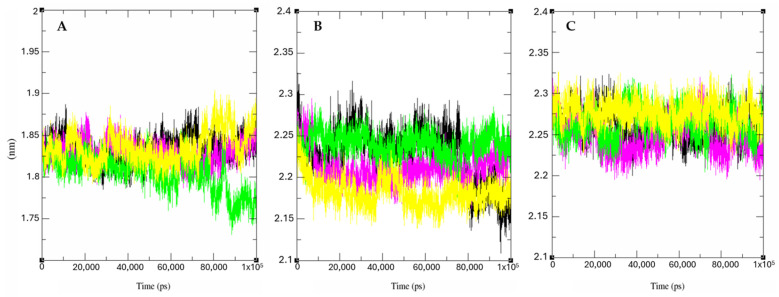
The radius of gyration (Rg). (**A**) Spike (6M0J); (**B**) 3CL^pro^ (6M2N); and (**C**) PL^pro^ (7TZJ). Complex protein bulbocapnine (**7**) (pink), complex protein reticuline (**2**) (green), complex protein inhibitor protein (yellow), and protein alone (black).

Additionally, we also analyzed the evolution of protein packing levels alone and in complex with Bulbocapnine, Reticuline, and the standard inhibitor ligand of each protein through radius of gyration (Rg) values. In summary, Rg provides an idea of how compact or extended a molecule is during a molecular dynamics simulation, providing information about the conformation, stability, interactions, and flexibility of the molecules under analysis. It was observed that the molecules, when coupled with proteins, did not exhibit significant variations compared to the protein alone (Figure 16). This indicates that the complexes are stable with low fluctuations in the tertiary structure of the enzymes.

The search for therapeutic alternatives to COVID-19 using natural products presents a complex challenge that requires innovative and integrated strategies. The main obstacles include the need for high-level biosafety laboratories to handle SARS-CoV-2, high costs associated with consumables and equipment, and rapid mutagenicity of the virus, which favors the spread of new variants. Although in vitro assays targeting viral structural proteins and key enzymes have advanced significantly, these approaches remain financially demanding and limited in their ability to replicate the complexity of in vivo environments. In parallel, computational approaches, such as molecular docking, provide valuable insights but face restrictions when applied to complex matrices, such as crude extracts, due to the high demand for computational resources and specialized infrastructure. In this context, the present study stands out for applying a robust multidisciplinary workflow that combines advanced experimental and computational techniques, such as chemometric analyses, molecular docking, and molecular dynamics, integrated with in vitro assays. This approach enables the efficient identification of bioactive compounds with antiviral potential by optimizing resources and reducing costs in the discovery of new molecules. The integrated use of experimental and computational data generates accurate predictive models, which can help direct efforts toward compounds with greater potential for viral inhibition. However, it is essential to acknowledge the study’s limitations. The absence of in vivo models to validate the therapeutic effects observed in in vitro assays constitutes a significant restriction, as it limits the extrapolation of results to more complex physiological conditions. Moreover, despite the rigor of applying computational tools, their predictive nature requires additional experimental validation, particularly regarding the bioavailability, metabolism, and toxicity of the identified compounds. Overcoming these limitations will require future studies involving in vivo assays, expansion of chemical libraries, and refinement of existing computational approaches. In light of these considerations, the present study offers significant methodological advancement by proposing an integrated and efficient workflow for discovering antiviral compounds from natural products. The combination of predictive chemometric models with targeted experimental validation expands the possibilities of identifying bioactive compounds with antiviral properties, optimizing resource use, and accelerating the discovery process. The results obtained contribute to advancing research in the field of natural antivirals and establishing a solid foundation for developing innovative and potentially patentable methods that will support future investigations in combating COVID-19 and other emerging viral pathogens.

## 3. Materials and Methods

### 3.1. Plant Material

Leaves of *S. ficoides*, *S. decipiens*, *S. glycycarpa*, *S. reginae*, and *S. cymosa* were collected from Reserva Adolpho Ducke/Manaus/Brazil (59°52′40″ and 59°52′00″ W, 03°00′00″ and 03°08′00″ S) [83], in August 2015. Voucher specimens are deposited at the Instituto Nacional de Pesquisas da Amazônia (INPA) herbarium (Manaus, Brazil) under the registration numbers INPA: 269731, 269734, 269732, 269735, and 269733, respectively. This work was authorized by the Directing Council of Genetic Heritage (Conselho de Gestão do Patrimônio Genético, CGEN) under authorization A3C04CB.

Dried and ground leaves were extracted by percolation with ethanol 96° GL. Part of the CEE was partitioned between water−methanol 7:3 (*v*/*v*) and HEX, DCM, EtOAc, and BuOH, affording their respective extracts. This method was performed as described by Leal et al. [31].

### 3.2. Functional Biological Assays

#### 3.2.1. Lumit™ RBD:ACE2 Interaction ASSAYS

SARS-CoV-2 Spike protein and human ACE2 complex formation were assessed using Lumit™ SARS-CoV-2 Spike RBD:ACE2 immunoassay (Promega, Madison, WI, USA). For this, 1 mg of each sample (*Siparuna* extracts) was separately solubilized in DMSO (dimethyl sulfoxide—Sigma-Aldrich, St. Louis, MO, USA) to a final concentration of 10 mg⋅mL^−1^. The solutions were centrifuged (5804 R—Eppendorf, Hamburg, Germany) for 5 min at 2500 RPM, and the supernatants were collected.

Briefly, the samples were added to a white 96-well plate to a final concentration of 250 μg·mL^−1^, followed by the addition of RBD (7.5 nM) and brief incubation (30 min). After adding ACE2 (7.5 nM), detection was carried out using secondary antibodies coupled to light particles after a 60 min incubation. The generated luminous signal was recorded in a SpectraMax M5 (Molecular Devices, San Jose, CA, USA) microplate reader at 500 ms of exposure as Relative Light Units (RLU). Percent inhibition was determined considering the total RLU resulting from successful RBD:ACE2 interaction (positive control), according to the following formula:

**Complex formation** (%) = [(treatment RLU − negative control 2)/total RLU in the positive control] × 100

The maximum interaction between RBD and ACE2 in the presence of the vehicle (DMSO) was used as the positive control for this assay. The negative controls comprised extracts with the detection reagents (negative control 1) and the detection reagents alone as a blank (negative control 2). The results are expressed as the means of three independent experiments.

#### 3.2.2. FRET Protease Assays with SARS-CoV-2 r3CL^pro^ and rPL^pro^

Recombinant SARS-CoV-2 3CL^pro^ and PL^pro^ enzymes expressed in *E. coli* BL21(DE3)pLysS and BL21(DE3) cells, respectively, were employed in a fluorescence resonance energy transfer (FRET) assay. The extracts were tested in triplicate at 100 µg/mL to evaluate the maximal inhibitory effect of each extract. For 3CL^pro^, the enzyme concentration was maintained at 1.5 μM, and the mixture was incubated with 5 mM NaCl, 20 mM Tris-HCl (pH 8.0), and 5 mM DTT (Merck, Darmstadt, Germany—97%, CAS 3483-12-3) for 15 min at 37 °C. For PL^pro^, a similar protocol was followed, but with the enzyme concentration set at 1 μM and reaction buffer containing 150 mM NaCl, 20 mM Tris-HCl (pH 8.0), and 5 mM DTT.

The reaction was initiated by adding the substrate (50 µM dissolved in DMSO). The customized fluorogenic substrates used were DABCYL-Ala-Val-Leu-Gln↓Ser-Gly-Phe-Arg-Lys-EDANS for 3CL^pro^ and DABCYL-Ala-Leu-Lys-Gly↓Gly-Lys-Iso-Val-EDANS for PL^pro^ (GenScript, Piscataway, NJ, USA, >95%). Fluorescence was measured using a SpectraMax M5 multi-mode microplate reader (Molecular Devices, San Jose, CA, USA). EDANS fluorescence emission was monitored at λexc = 330 nm and λem = 490 nm at 37 °C for 45 min. Fluorescence data (RFU) were converted to substrate cleavage-specific activity based on a previously calculated fluorescence conversion factor (FEC) for the EDANS-DABCYL fluorophore pair. The maximum enzyme activity was measured using the vehicle (DMSO), and these values were used to calculate the inhibition percentages of the test extracts. Additionally, the fluorescence profiles of the test extracts were assessed under the same assay conditions (dissolved in DMSO, buffer, and substrate). This step was essential to confirm the reliability of the inhibition results. Enzyme activity was also evaluated using the commercial inhibitors CG-376 for 3CL^pro^ and GRL-0617 for PL^pro^ (Supplementary Materials Figures S8 and S9).

### 3.3. UHPLC-MS/MS Analysis

The extracts (2 mg⋅mL^−1^) were analyzed in UHPLC Dionex Ultimate 3000 (ThermoFischer Scientific, Waltham, MA, USA) coupled to LCQfleet (ThermoFischer Scientific, Waltham, MA, USA). The analyses were performed using an Acquity BEH C18 column (Waters Corporation, Milford, MA, USA—1.7 μm, 2.1 × 100 mm, 100 Å) with a flow rate of 0.45 mL⋅min^−1^ according to the elution gradient: (0 min, 5%B), (5 min, 5%B), (25 min, 100%B), (30 min, 100%B), (31 min, 5%B), (36 min, 5%B).

The mass spectrometer, equipped with atmospheric pressure chemical ionization (APCI) and electrospray ionization (ESI), was operated in positive and negative ionization modes. MS spectra were acquired in the range of *m*/*z* 100–1000, and the normalized collision energy of the collision-induced dissociation (CID) cell was set at 35 eV. LC-MS/MS analysis was conducted on 25 extracts (five extracts of each *Siparuna* spp.) in three replicates (totaling 300 analyses).

### 3.4. Computational Analysis and Annotation of Molecules

The data acquired from LC-MS/MS analyses were converted to the mzML format using specific parameters, including subset, msLevel 1–2, and positive or negative polarity. Additionally, Peak Picking with the vendor algorithm (msLevel 1–2) in the Proteowizard—MSconvert version 3.02 tool (ProteoWizard Software Foundation, Palo Alto, CA, USA) was applied. Subsequently, MZmine v.2.53 (University of Helsinki, Helsinki, Finland) was employed for data processing, with mass detection adjusted based on signal intensity and quality obtained from different analysis methods (ESI or APCI [+/−]). The ADAP Chromatogram Builder algorithm facilitated mass chromatogram construction and wavelet deconvolution. Isotopes were removed using the Isotopic Peaks Grouper, with the most intense isotope chosen as the representative, and alignment was performed using the join aligner.

Following this, both processed chemical data (*m*/*z* ions and retention time) and biological data (% inhibition) were exported to UnscramblerX software version 10.4 (CAMO Software, Oslo, Norway). The aim of this study was to predict potential active compounds inhibiting the Spike:ACE2 interaction and the proteolytic activities of r3CL^pro^ and rPL^pro^. Chemometric analyses were performed with low-level data fusion (concatenated ESI and APCI [+/−] data) and a Partial Least Squares Regression model correlated LC-MS (data fusion) with inhibitory activities. For PLS regression, the samples were classified into two groups (binary values) based on average activity values: active samples (≥50% inhibition—[1]) and non-active samples (<50% inhibition—[−1]). The NIPALS algorithm was used to build the PLS model, and the cross-validation method used was Leave-One-Out. The ions indicated in the PLS prediction models were obtained from a custom data library.

### 3.5. In Vitro Studies in Calu-3 Cells

#### 3.5.1. Cytotoxicity ASSAY

The Calu-3 cell line, originating from human bronchial adenocarcinoma and supplied by the Farmanguinhos platform RPT11M, was propagated in 96-well plates (1.5 × 10^4^ cells/well). These cells were exposed to 25 different extracts at a concentration of 200 µg·mL^−1^ for a duration of 72 h to evaluate cytotoxicity using the CytoTox 96^®^ Non-Radioactive Colorimetric Assay Kit (Promega, Madison, WI, USA). This assay quantifies the activity of the enzyme Lactate Dehydrogenase (LDH) as per the guidelines provided by the manufacturer, replicating previously conducted procedures [24]. Briefly, cells in the control group (non-treated) were lysed using the Lysis Solution included in the kit. Subsequently, the plate was centrifuged at 400× *g* for three minutes, after which 50 μL of the supernatant was carefully transferred to another plate. CytoTox 96^®^ reagent was then added to this supernatant, and the mixture was incubated for 30 min at room temperature, shielded from light. Following incubation, the Stop Solution was administered, and absorbance was recorded at 490 nm using a spectrophotometer (Loccus, São Paulo, Brazil). The results were expressed as a percentage of viable cells based on the following calculation:

$$\;Cell\;Viability\;=100-([\left(DMSO\;-\;treatment\right)\times 100])/(Lysis\;Control)\;\;$$

For this investigation, the extracts were first dissolved in 100% dimethyl sulfoxide (DMSO) and then further diluted in Dulbecco’s Modified Eagle Medium (DMEM—Gibco, Waltham, MA, USA). The concentrations of DMSO were carefully maintained below 1% (*v*/*v*) to minimize any impact on cellular proliferation and viability [84]. The graph was constructed and analyzed using GraphPad Prism software, version 10.2.1 (GraphPad Software, San Diego, CA, USA), and represents the mean of the data performed with at least 3 technical replicates.

#### 3.5.2. Anti-SARS-CoV-2 Assay

To assess the antiviral properties of the 25-extracts, Calu-3 cells were prepared as previously described and infected with SARS-CoV-2 virus, B1 lineage (GenBank MT710714, SisGen AC58AE2), at a multiplicity of infection (MOI) of 0.01. The infection process involved incubating the cells at 37 °C and 5% CO_2_ for one hour to enable viral adsorption and entry. Post-viral entry, the extracts were administered at 50 µg·mL^−1^ over 24 h. After the treatment period, supernatants were collected to measure viral titers using the plaque assay technique. This involved inoculating Vero E6 (African green monkey kidney cells, ATCC CRL-1586) cells at 1.0 × 10^4^ cells/well, exposing them to serial dilutions (from 1:200 to 1:25,600) of the supernatants for one hour, and overlaying with 50 μL of a 2.4% Carboxymethylcellulose medium, which includes DMEM-High Glucose 10×, 2.4% Carboxymethylcellulose, 2% Fetal Bovine Serum, and 1% Penicillin/Streptomycin. After a 72-h incubation under consistent conditions, cells were fixed with 100 μL of 4% formalin for three hours and stained with 0.04% crystal violet for one hour. Plaque-forming units (PFU) were counted, and viral titers were expressed as PFU/mL. The viral titers in the supernatants of the infected and treated cells were compared with those of the untreated control cells and expressed as a percentage of viral replication inhibition. Notably, all experimental procedures and viral handling were conducted in a biosafety level-3 laboratory (BSL-3) in strict accordance with the WHO guidelines [85]. The graph was constructed and analyzed using GraphPad Prism software, version 10.2.1, and represents the mean of the data, performed with at least 3 technical replicates.

### 3.6. In Silico Studies

#### 3.6.1. ADMET Predictions

Prediction of absorption, distribution, metabolism, and excretion (ADME) parameters was calculated using the Swiss ADME open-access web tool (Swiss Institute of Bioinformatics, Lausanne, Switzerland) [86,87]. The toxicity prediction was performed in DataWarrior v. 4.7.2 software called OpenMolecules (Actelion Pharmaceuticals Ltd., Allschwil, Switzerland) [88,89].

#### 3.6.2. Molecular Docking

Six proteins associated with SARS-CoV-2 were sourced from the Protein Data Bank (PDB) library [90,91]. Specifically, these proteins included those corresponding to the Spike protein (PDB 3SCI and 6M0J) [72,73], 3CL^pro^ (PDB 6M2N and 7B3E) [74,75], and PL^pro^ (PDB 7LBR and 7TZJ) [76,77]. The active site regions were defined based on the information available in the PDB library reference articles [92] cited above. In cases where enzymes did not have co-crystallized ligands, the active sites were defined based on ligand-protein coordinates.

All compounds were prepared, drawn using Marvin Sketch v19.18 (ChemAxon, Budapest, Hungary—https://chemaxon.com/marvin, accessed on 1 July 2024), and saved as .sdf files. Standardization was carried out using Standardizer v21.2.0 (Standardizer v21.2.0—https://chemaxon.com/standardizer, accessed on 1 July 2024), involving the addition of hydrogen atoms, aromatic ring standardization, salt removal, and structure conversion to 3D. Molecular docking was then performed, involving the removal of water molecules and the creation of a “template” between the macromolecule and co-crystallized ligand to delineate the active site, followed by the insertion of test molecules.

For enzymes lacking co-crystallized ligands, like the Spike protein (PDB 3SCI and 6M0J), the active site was identified using Molecular Pocket Predictors, such as the Bite Net Platform—Skoltech iMolecule, 2022 (Skoltech, Skolkovo Institute of Science and Technology, Moscow, Russia) [78,93,94,95]. Control compounds, including remdesivir [79], were used for validation.

The Molegro Virtual Docker v.6.0.1 (MVD) software (CLC bio, QIAGEN, Aarhus, Denmark) was employed with its default parameters. The active site was defined using the complexed ligand. Subsequently, the compounds were imported for analysis of system stability based on interactions identified with the enzyme active site, referencing the energetic value of the MolDock Score algorithm [96,97,98].

The MolDock SE and PLANTS (Simplex Evolution) algorithms were utilized with the following parameters: a total of 30 runs, a maximum of 3000 interactions, a population of 50 individuals, 2000 minimization steps for each flexible residue, and 2000 global minimization steps per run. The MolDock Score, PLANTS Score, and Rerank Score scoring functions were applied to calculate the docking energy values. A GRID was established at 0.3 Å, and the search sphere had a radius of 15 Å. The evaluation of ligand energy encompassed the analysis of internal electrostatic interactions, internal hydrogen bonds, and sp2-sp2 torsions. Discovery Studio Visualizer v20.1. Software (BIOVIA, Dassault Systèmes, San Diego, CA, USA) [99] was employed to visualize the interactions and generate molecular docking figures.

#### 3.6.3. Docking Consensus

A consensus analysis was performed using three scoring functions—MolDock Score, Rerank Score, and PLANTS Score to reduce false positives [100,101]. The affinity results from each function were included in the consensus calculations. For each scoring function, the relative score (*p*) was calculated by dividing each compound’s score by the score of the compound with the lowest energy, as shown in Equation (1).

(1) Prob=((E Lig))/((EMin Lig))

Probability (*Prob*) represents the probability of activity, while *E Lig* is the calculated energy for each ligand in the docking simulation. *EMin Lig* corresponds to the lowest energy obtained among the analyzed ligands. In the consensus analysis, the overall average is calculated for all biomarkers under study. Thus, the probability for each compound is obtained as an overall average of the probabilities for each biomarker. After calculating these probabilities in each scoring function analyzed, the total probability (*P*) is determined by summing the probabilities and dividing by the total number of observations (*n*)—that is, the number of scoring functions considered—as per Equation (2).

(2) Ptotal=((P MolDock Score+P Rerank Score+P Plants Score))/(n)

After calculating the total probability for each of the PDB structures used, the probability for the target under study (*P* Enzyme—Equation (3)) was obtained by summing the total probabilities and dividing by the number of 3D structures considered.

(3) P enzyme=((Ptotal 01+Ptotal 02))/n

This analysis was conducted to offer a more thorough understanding of the compounds’ affinity for the target, as the total probability value incorporated multiple crystallographic structures.

#### 3.6.4. Molecular Dynamics

The Gromacs 5.0 software (Royal Institute of Technology, Stockholm, Sweden) [102,103] was used to perform molecular dynamics simulations to evaluate the stability of interactions between proteins and ligands. The topology of the binder was prepared through the topology generator PRODRG [104], applying the same force field as Gromacs for the protein, GROMOS43a1. For the molecular dynamics simulation, the SPC model of the extended point load was used in a dodecahedron box [105].

For electrical neutralization of the system, chloride and sodium ions were added, and the minimization of the system was also balanced at a temperature of 300 K by the V-resizing algorithm at 100 ps NVT set (constant number of particles, volume, and temperature), followed by equilibrium at 1 atm of pressure with the Parrinello–Rahman algorithm as set NPT (constant number of particles, pressure, and temperature) to 100 ps. DM simulations were performed for 50,000,000 cycles at 50 ns.

To determine the stability of the structure and if the complex was stable near the experimental framework, the mean square root displacement (RMSD) of all heavy atoms was calculated relative to the starting structures. Residual fluctuations (RMSF) were also analyzed to understand the role of residues near the receptor-binding site. To verify the interaction energy of the protein–ligand complex, we calculated the short-range Coulombic interaction energy and the Lennard-Jones short-range energy for 50 ns. The Root Mean Square Deviation (RMSD) and Root Mean Square Fluctuation (RMSF) plots were generated in the Grace [106] software, and the protein and ligands were visualized with UCSF Chimera (Resource for Biocomputing, Visualization, and Informatics, University of California, San Francisco, CA, USA) [107].

## 4. Conclusions

In the present study, the established workflow combining biological and chemical screening of crude plant extracts, along with computational methods, enabled the accelerated prediction of compounds with potential anti-SARS-CoV-2 activity from the Siparunaceae family. Biological assays conducted with extracts from the leaves of *Siparuna cymosa*, *S. decipiens*, *S. ficoides*, *S. glycycarpa*, and *S. reginae* on different SARS-CoV-2 targets corroborate previous studies reporting the antiviral potential of this genus. The results identified extracts with significant multitarget activity, particularly in inhibiting the RBD:ACE2 interaction and the activities of 3CL^pro^ and PL^pro^ proteases.

The integration of chemometric simulations and custom library searches revealed the presence of seven isoquinoline alkaloids previously isolated and/or identified in *Siparuna* species, with an emphasis on *S. decipiens*, *S. glycycarpa*, and *S. cymosa*. Among these, two compounds stood out: bulbocapnine (an aporphine alkaloid) and reticuline (a benzylisoquinoline alkaloid), which demonstrated superior binding potential to SARS-CoV-2 target proteins in molecular docking studies. Notably, the ADME results indicated that none of the molecules violated Lipinski’s rules, although coclaurine exhibited potential tumorigenicity.

Molecular dynamics simulations highlighted bulbocapnine for its exceptional stability in protein−ligand interactions, making it a promising multitarget inhibitor of coronaviruses. This compound emerged with the highest activity potential across all constructed chemometric models, along with favorable ADMET profiles when compared to the respective positive controls (protein inhibitors). Importantly, this study is the first to report bulbocapnine as a potential anti-SARS-CoV-2 agent.

Additionally, bulbocapnine was observed to be relatively more abundant in the dichloromethane extracts of *S. glycycarpa*, positioning this extract as a viable option for the purification of this compound. These findings support further exploration, isolation, and in-depth testing of bulbocapnine and related alkaloids, contributing to the ongoing search for new drug candidates derived from natural products as alternative treatments for COVID-19. In conclusion, bulbocapnine’s stable and enduring interactions with multiple SARS-CoV-2 target proteins underscore its significant therapeutic potential.

## Figures and Tables

**Table 1:** LC-MS/MS data of predicted ions and annotation of the proposed compounds.

ID	Rt (min)	Molecular Formula	[M + H]^+^ *m*/*z*	Source	MS/MS (MS^2^)	Proposed Compound	Ref.
**1**	9.0	C_17_H_19_NO_3_	286	ESI	269 (-NH_3_), 237 (-NH_3_-CH_3_OH), 209 (-NH_3_-CH_3_OH-CO), 191 (-NH_3_-CH_3_OH-CO-H_2_O)	coclaurine	[31,40]
**2**	10.1	C_19_H_23_NO_4_	330	APCI	299 (-CH_3_NH_2_), 281 (-CH_3_NH_2_-H_2_O), 267 (-CH_3_NH_2_-CH_3_OH), 235 (-CH_3_NH_2_-2xCH_3_OH), 207 (-CH_3_NH_2_-2xCH_3_OH-CO)	reticuline	[31,40]
**3**	10.3	C_19_H_21_NO_4_	328	ESI/APCI	297 (-CH_3_NH_2_), 282 (-CH_3_NH_2_-CH_3_), 265 (-CH_3_NH_2_-CH_3_OH), 237 (-CH_3_NH_2_-CH_3_OH-CO)	boldine	[40]
**4**	10.6	C_18_H_19_NO_4_	314	APCI	297 (-NH_3_), 282 (-CH_3_OH), 265 (-NH_3_-CH_3_OH)	laurolitsine	[31]
**5**	10.8	C_17_H_17_NO_2_	268	ESI	251 (-NH_3_), 219 (-NH_3_-CH_3_OH), 191 (-NH_3_-CH_3_OH-CO)	asimilobine	[43]
**6**	11.2	C_18_H_17_NO_4_	312	ESI	295 (-NH_3_), 280 (-CH_3_OH), 263 (-NH_3_-CH_3_OH), 254 (-CH_2_O-CO)	actinodaphnine	[31,44]
**7**	11.6	C_19_H_19_NO_4_	326	ESI	295 (-CH_3_NH_2_), 280 (-CH_3_NH_2_-CH_3_), 263 (-CH_3_NH_2_-CH_3_OH), 235 (-CH_3_NH_2_-CH_3_OH-CO)	bulbocapnine	[31]

**Table 2:** Predicted interactions with cytochrome P450 (CYP) for the seven compounds.

ID	CYP				
	CYP1A2	CYP2C19	CYP2C9	CYP2D6	CYP3A4
**1**	No	No	No	Yes	No
**2**	No	No	No	Yes	No
**3**	Yes	No	No	Yes	Yes
**4**	Yes	No	No	Yes	Yes
**5**	Yes	No	No	Yes	Yes
**6**	Yes	No	No	Yes	Yes
**7**	Yes	Yes	Yes	Yes	Yes

**Table 3:** Information about the target proteins of SARS-CoV-2, their respective ligands, and the RMSD values obtained from the redocking procedure.

Protein ID	Ligand	Redocking, RMSD Value (Å)	Reference
3CL^pro^ (6M2N)	5,6,7-trihydroxy-2-phenyl-4H-chromen-4one	0.1019	[74]
3CL^pro^ (7B3E)	3,5,7-trihydroxy-2-(3,4,5-trihydroxyphenyl)-4H-chr- romen-4-one	0.1583	[75]
PL^pro^ (7LBR)	5-[(azetidine-3-yl)amino]-N-[(1R)-1-{3-[5-({[(1S,3R)-3-hydroxycyclopentyl]amino}methyl)thiophe-n-2-yl]phenyl}ethyl]-2-methylbenzamide	1.1780	[76]
PL^pro^ (7TZJ)	–[(3-fluorophenyl)methyl]-1-[(1R)-1-naphtalen-1-ylethyl]piperidine-4-carboxamide	1.2253	[77]

**Table 4:** Score values and probability of activity of the test compounds on the targets of SARS-CoV-2.

ID	Spike Protein			3CL^pro^			PL^pro^		
	*p* Total (3SCI)	*p* Total (6M0J)	(*p*) Enzyme	*p* Total (6M2N)	*p* Total (7B3E)	(*p*) Enzyme	*p* Total (7LBR)	*p* Total (7TZJ)	(*p*) enzyme
**1**	0.68	0.76	0.72	0.87	0.72	0.80	0.62 *	0.76	0.69 *
**2**	0.73	0.66	0.69	0.89	0.89 *	0.89 *	0.54	0.66	0.60
**3**	0.72	0.69	0.71	0.89	0.79	0.84	0.57	0.64	0.61
**4**	0.64	0.63	0.64	0.71	0.45	0.58	0.54	0.65	0.60
**5**	0.77 *	0.69	0.73 *	0.85	0.88	0.87	0.61	0.72	0.66 *
**6**	0.74	0.69	0.72	0.89	0.72	0.81	0.56	0.76	0.65
**7**	0.75	0.78 *	0.77 *	0.94 *	0.87	0.90 *	0.45	0.84 *	0.65
PDB/PC	1.0	0.97	0.98	0.85	0.60	0.72	1.0	0.99	0.99

PDB: Ligand PDB; PC: Positive Control; * In bold is the lowest score.

## Data Availability

The data presented in this study are available on request from the corresponding authors.

## Supplementary Materials

The following supporting information can be downloaded at https://www.mdpi.com/article/10.3390/ijms26020633/s1. Figure S1: ESI-MS/MS spectrum of compound **1** at *m*/*z* 286 [M+H]^+^ (coclaurine); Figure S2: APCI-MS/MS spectrum of compound **2** at *m*/*z* 330 [M+H]^+^ (reticuline); Figure S3: ESI-MS/MS spectrum of compound **3** at *m*/*z* 328 [M+H]^+^ (boldine); Figure S4: APCI-MS/MS spectrum of compound **4** at *m*/*z* 314 [M+H]^+^ (laurolitsine); Figure S5: ESI-MS/MS spectrum of compound **5** at *m*/*z* 268 [M+H]^+^ (assimilobine); Figure S6: ESI-MS/MS spectrum of compound **6** at *m*/*z* 312 [M+H]^+^ (actinodaphnine); Figure S7: ESI-MS/MS spectrum of compound **7** at *m*/*z* 326 [M+H]^+^ (bulbocapnine); Figure S8: Evaluation of the inhibitory activity of GC376 against 3CL^pro^ at concentrations of 100, 10, 1, 0.1, and 0.01 µM; Figure S9: Evaluation of the inhibitory activity of GRL-0617 against 3CL^pro^ at concentrations of 100, 10, 1, 0.1, and 0.01 µM; Table S1: ADMET properties predictions by SwissModel; Table S2: Score values and probability of activity of the test compounds on the target 3CL^pro^ (PDB: 6M2N); Table S3: Score values and probability of activity of the test compounds on the target 3CL^pro^ (PDB: 7B3E); Table S4: Score values and probability of activity of the test compounds on the target PL^pro^ (PDB: 7LBR); Table S5: Score values and probability of activity of the test compounds on the target PL^pro^ (PDB: 7TZJ); Table S6: Score values and probability of activity of the test compounds on the target Spike (PDB: 3SCI); Table S7: Score values and probability of activity of the test compounds on the target Spike (PDB: 6M0J).
