# Supplementary material for: Predicting the Anti-SARS-CoV-2 Potential of Isoquinoline Alkaloids from Brazilian Siparunaceae Species Using Chemometric Tools
Source: Int J Mol Sci. 2025 Jan 13;26(2):633. doi: 10.3390/ijms26020633 (PMC11765762; doi:10.3390/ijms26020633)
Supplement: Supplementary file 1 [file ijms-26-00633-s001.zip › ijms-3375595-supplementary.pdf]

Supplementary material

Predicting the Anti-SARS-CoV-2 Potential of Isoquinoline Alkaloids from Brazilian Siparunaceae Species Using Chemometric Tools

Brendo Araujo Gomes [1,2,†], Diégina Araújo Fernandes [3,†], Simony Carvalho Mendonça [2,4], Mariana Freire Campos [1,2], Thamirys Silva da Fonseca [2,4], Larissa Esteves Carvalho Constant [5], Natalia Ferreira de Sousa [6], Renata Priscila Barros de Menezes [6], Beatriz Albuquerque Custódio de Oliveira [7], Stephany da Silva Costa [7], Giovanna Barbosa Frensel [7], Alice Santos Rosa [8,9], Thamara Kelcya Fonseca Oliveira [8,9], Amanda Resende Tucci [8,9], Júlia Nilo Henrique Lima [8], Vivian Neuza Santos Ferreira [8], Milene Dias Miranda [8,9], Diego Allonso [5,7,10], Marcus Tullius Scotti [6,10], Suzana Guimarães Leitão [1,2,4,*] and Gilda Guimarães Leitão [3,4,*]

1. Programa de Pós-Graduação em Biotecnologia Vegetal e Bioprocessos, Centro de Ciências da Saúde, Universidade Federal do Rio de Janeiro, Rio de Janeiro 21941-902, RJ, Brazil; brendoo.bc@ufrj.br (B.A.G.); ccamposmariana@ufrj.br (M.F.C.)
2. Departamento de Produtos Naturais e Alimentos, Faculdade de Farmácia, Universidade Federal do Rio de Janeiro, Rio de Janeiro 21941-902, RJ, Brazil; sy2802@ufrj.br (S.C.M.); thamirysfonseca@ufrj.br (T.S.d.F.)
3. Instituto de Pesquisas de Produtos Naturais, Universidade Federal do Rio de Janeiro, Rio de Janeiro 21941-902, RJ, Brazil; diegina@ufrj.br (D.A.F.); ggleitao@ippn.ufrj.br (G.G.L.)
4. Programa de Pós-Graduação em Ciências Farmacêuticas, Faculdade de Farmácia, Universidade Federal do Rio de Janeiro, Rio de Janeiro 21941-902, RJ, Brazil
5. Programa de Pós-Graduação em Ciências Biológicas, Instituto de Biofísica Carlos Chagas Filho, Universidade Federal do Rio de Janeiro, Rio de Janeiro 21941-590, RJ, Brazil; larissaestevescarvalho@gmail.com (L.E.C.C.); diegoallonso@pharma.ufrj.br (D.A.)
6. Programa de Pós-Graduação em Produtos Naturais e Sintéticos Bioativos, Universidade Federal da Paraíba, João Pessoa 58015-970, PB, Brazil; nataliafsousa@ltf.ufpb.br (N.F.d.S.); renatabarros@ltf.ufpb.br (R.P.B.d.M.); mtscotti@ccae.ufpb.br (M.T.S.)
7. Laboratório de Biotecnologia e Bioengenharia Estrutural, Universidade Federal do Rio de Janeiro, Centro de Ciências da Saúde, Bloco G, Rio de Janeiro 21941-902, RJ, Brazil; beatrizalbuquerquecp2@gmail.com (B.A.C.d.O.); stephanycosta@biof.ufrj.br (S.d.S.C.); gfrensel@peq.coppe.ufrj.br(G.B.F.)
8. Laboratório de Morfologia e Morfogênese Viral, Oswaldo Cruz Institute, Oswaldo Cruz Foundation, Rio de Janeiro 21041-250, RJ, Brazil; alicerosa@aluno.fiocruz.br (A.S.R.); thamarafonseca@ufmg.br (T.K.F.O.); artucci.bio@gmail.com(A.R.T.); julianilo@ufrj.br (J.N.H.L.); vivian.ferreira@ioc.fiocruz.br (V.N.S.F.); mmiranda@ioc.fiocruz.br (M.D.M.)
9. Programa de Pós-Graduação em Biologia Celular e Molecular, Instituto Oswaldo Cruz, Fundação Oswaldo Cruz, Rio de Janeiro 21041-250, RJ, Brazil
10. Departamento de Biotecnologia Farmacêutica, Faculdade de Farmácia, Universidade Federal do Rio de Janeiro, Rio de Janeiro 21941-902, RJ, Brazil

* Correspondence: sgleitao@pharma.ufrj.br (S.G.L.), ggleitao@ippn.ufrj.br (G.G.L.)

† These authors contributed equally to this work.

Summary

Figures

Figure S1. ESI-MS/MS spectrum of compound **1** at *m/z* 286 $[M+H]^+$ (coclaurine)

Figure S2. APCI-MS/MS spectrum of compound **2** at *m/z* 330 $[M+H]^+$ (reticuline)

Figure S3. ESI-MS/MS spectrum of compound **3** at *m/z* 328 $[M+H]^+$ (boldine)

Figure S4. APCI-MS/MS spectrum of compound **4** at *m/z* 314 $[M+H]^+$ (laurolitsine)

Figure S5. ESI-MS/MS spectrum of compound **5** at *m/z* 268 $[M+H]^+$ (assimilobine)

Figure S6. ESI-MS/MS spectrum of compound **6** at *m/z* 312 $[M+H]^+$ (actinodaphnine)

Figure S7. ESI-MS/MS spectrum of compound **7** at *m/z* 326 $[M+H]^+$ (bulbocapnine)

Figure S8. Evaluation of the inhibitory activity of GC376 against $3CL^{pro}$ at concentrations of 100, 10, 1, 0.1, and 0.01 μM.

Figure S9. Evaluation of the inhibitory activity of GRL-0617 against $3CL^{pro}$ at concentrations of 100, 10, 1, 0.1, and 0.01 μM.

Tables

Table S1. ADMET properties predictions by SwissModel.

Table S2: Score values and probability of activity of the test compounds on the target $3CL^{pro}$ (PDB: 6M2N).

Table S3: Score values and probability of activity of the test compounds on the target $3CL^{pro}$ (PDB: 7B3E).

Table S4: Score values and probability of activity of the test compounds on the target PL^{pro} (PDB: 7LBR).

Table S5: Score values and probability of activity of the test compounds on the target PL^{pro} (PDB: 7TZJ).

Table S6: Score values and probability of activity of the test compounds on the target Spike (PDB: 3SCI).

Table S7: Score values and probability of activity of the test compounds on the target Spike (PDB: 6M0J).

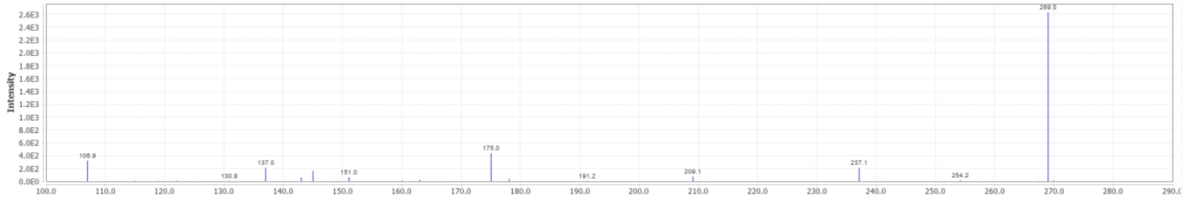

Figure S1. ESI-MS/MS spectrum of compound **1** at *m/z* 286 [M+H]$^+$ (coclaurine)

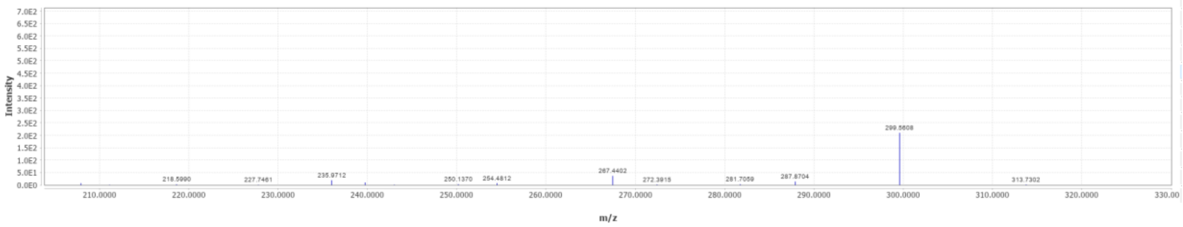

Figure S2. APCI-MS/MS spectrum of compound **2** at *m/z* 330 [M+H]$^+$ (reticuline)

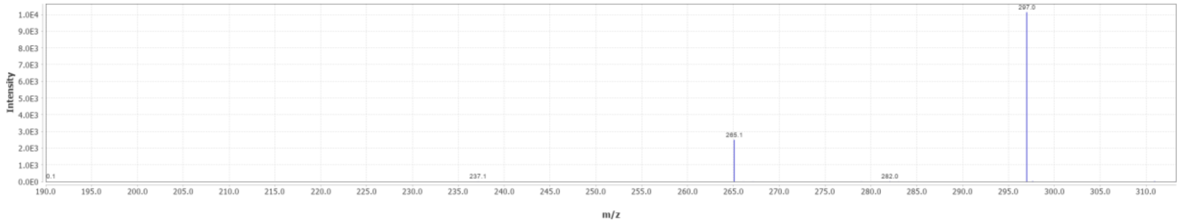

Figure S3. ESI-MS/MS spectrum of compound **3** at *m/z* 328 [M+H]$^+$ (boldine)

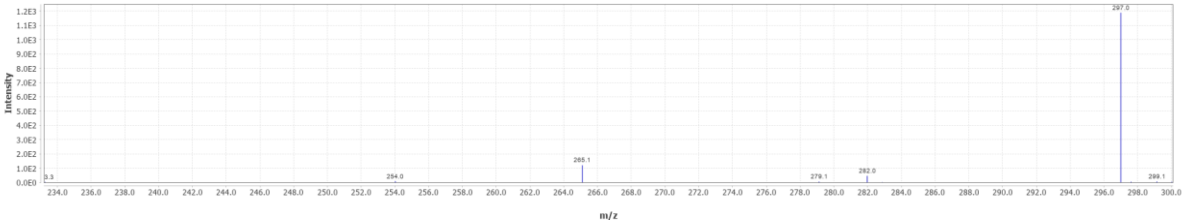

Figure S4. APCI-MS/MS spectrum of compound **4** at *m/z* 314 [M+H]$^+$ (laurolitsine)

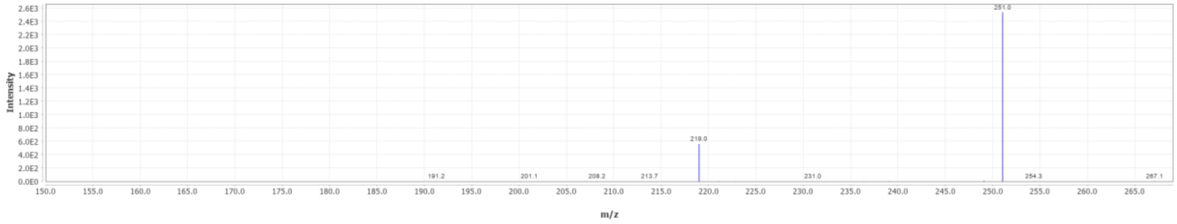

Figure S5. ESI-MS/MS spectrum of compound **5** at *m/z* 268 [M+H]$^+$ (assimilobine)

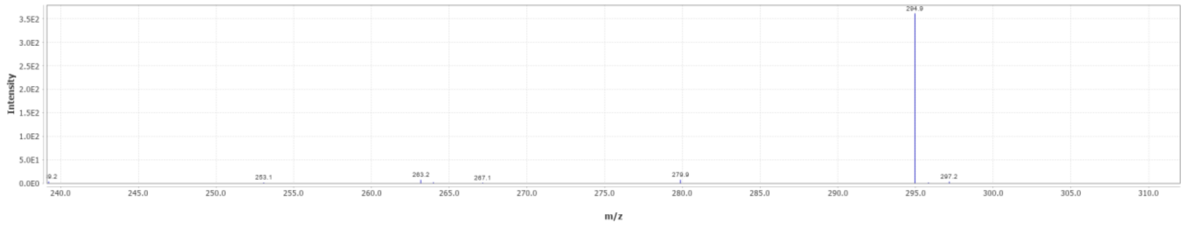

Figure S6. ESI-MS/MS spectrum of compound **6** at *m/z* 312 $[M+H]^+$ (actinodaphnine)

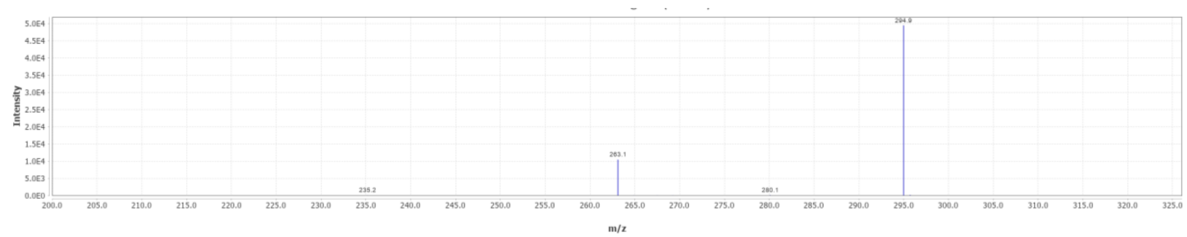

Figure S7. ESI-MS/MS spectrum of compound **7** at *m/z* 326 $[M+H]^+$ (bulbocapnine)

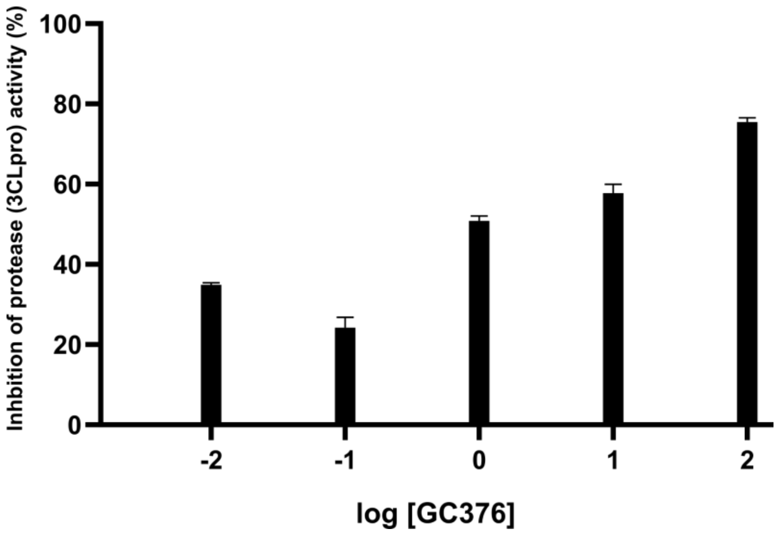

Figure S8. Evaluation of the inhibitory activity of GC-376 against $3CL^{pro}$ at concentrations of 100, 10, 1, 0.1, and 0.01 μM.

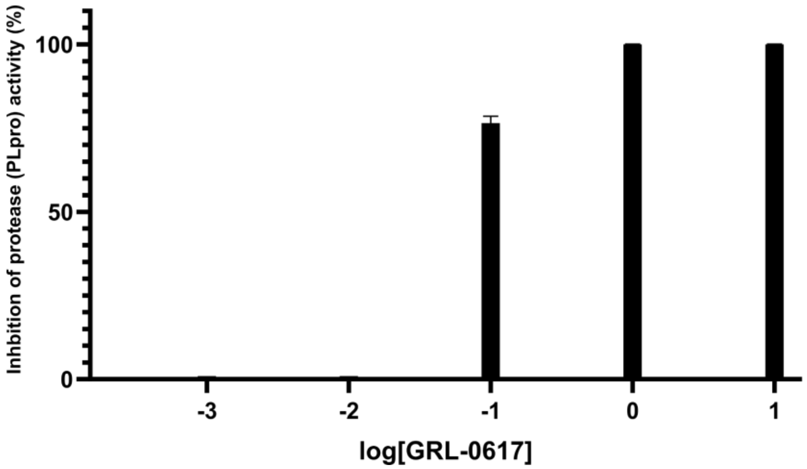

Figure S9. Evaluation of the inhibitory activity of GRL-0617 against 3CL^{pro} at concentrations of 10, 1, 0.1, 0.01 and 0.001 nM.

Table S1. ADMET properties predictions by SwissModel.

Compound		Actinodaphine	Boldine	Coclaurine	Laurolitsine	Assimilobine	Reticuline	Bulbocapnine
**Physicochemical properties**	Formula Molecular	$C_{18}H_{17}NO_4$	$C_{20}H_{23}NO_3$	$C_{17}H_{19}NO_3$	$C_{18}H_{19}NO_4$	$C_{17}H_{17}NO_2$	$C_{19}H_{23}$NO4	$C_{19}H_{19}NO_4$
	Molecular Weight	311.33	325.4	285.34	313.35	267.32	329.39	325.36
	#Heavy atoms	23	24	21	23	20	24	24
	#Aromatic heavy atoms	12	12	12	12	12	12	12
	Fraction Csp3	0.33	0.4	0.29	0.33	0.29	0.37	0.37
	#Rotatable bonds	1	2	3	2	1	4	1
	#H-bond acceptors	5	4	4	5	3	5	5
	#H-bond donors	2	1	3	3	2	2	1
	MR	88.65	98.95	85.62	91.1	82.59	97.01	93.55
	TPSA[2]	59.95	41.93	61.72	70.95	41.49	62.16	51.16
**Lipophilicity**	iLOGP	2.95	3.33	2.55	2.71	2.58	3.13	3.16
	XLOGP3	2.45	3.44	2.58	2.25	2.64	3.01	2.92

Table S1 (cont.) ADMET properties predictions by SwissModel.

Compound		Actinodaphine	Boldine	Coclaurine	Laurolitsine	Assimilobine	Reticuline	Bulbocapnine
**Lipophilicity**	WLOGP	1.83	2.76	1.83	1.82	2.11	2.18	2.18
	MLOGP	1.92	2.54	1.84	1.52	2.43	1.75	2.16
	Silicos-IT Log P	3.34	3.95	2.94	3.02	3.48	2.92	3.27
	Consensus Log P	2.5	3.2	2.35	2.26	2.65	2.6	2.74
**Water solubility**	ESOL Log S	-3.63	-4.26	-3.46	-3.45	-3.54	-3.88	-4
	ESOL Solubility (mg/ml)	7.23e-02	1.78e-02	9.91e-02	1.10e-01	7.73e-02	4.30e-02	3.25e-02
	ESOL Solubility (mol/l)	2.32e-04	5.46e-05	3.47e-04	3.51e-04	2.89e-04	1.30e-04	9.98e-05
	ESOL Class	S[3]	MS[4]	S	S	S	S	MS
	Ali Log S	-3.35	-4	-3.52	-3.38	-3.16	-3.98	-3.66
	Ali Solubility (mg/ml)	1.38e-01	3.24e-02	8.53e-02	1.32e-01	1.84e-01	3.45e-02	7.19e-02
	Ali Solubility (mol/l)	4.44e-04	9.97e-05	2.99e-04	4.21e-04	6.89e-04	1.05e-04	2.21e-04
	Ali Class	S	MS	S	S	S	S	S

Table S1 (cont.) ADMET properties predictions by SwissModel.

Compound		Actinodaphine	Boldine	Coclaurine	Laurolitsine	Assimilobine	Reticuline	Bulbocapnine
**Water solubility**	Silicos-IT LogSw	-5.13	-5.55	-4.94	-4.93	-5.39	-4.72	-4.79
	Silicos-IT Solubility (mg/ml)	2.32e-03	9.11e-04	3.25e-03	3.72e-03	1.09e-03	6.25e-03	5.26e-03
	Silicos-IT Solubility (mol/l)	7.46e-06	2.80e-06	1.14e-05	1.19e-05	4.06e-06	1.90e-05	1.62e-05
	Silicos-IT class	MS	MS	MS	MS	MS	MS	MS
**Pharmacokinetic properties**	GI[5] absorption	High	High	High	High	High	High	High
	BBB[6] permeant	Yes	Yes	Yes	Yes	Yes	Yes	Yes
	Pgp[7] substrate	Yes	Yes	Yes	Yes	Yes	Yes	Yes
	CYP1A2 inhibitor	Yes	Yes	No	Yes	Yes	No	Yes
	CYP2C19 inhibitor	No	No	No	No	No	No	Yes
	CYP2C9 inhibitor	No	No	No	No	No	No	Yes

Table S1 (cont.) ADMET properties predictions by SwissModel.

Compound		Actinodaphine	Boldine	Coclaurine	Laurolitsine	Assimilobine	Reticuline	Bulbocapnine
**Pharmacokinetic properties**	CYP2D6 inhibitor	Yes	Yes	Yes	Yes	Yes	Yes	Yes
	CYP3A4 inhibitor	Yes	Yes	No	Yes	Yes	No	Yes
	log Kp (cm/s)	-6.46	-5.84	-6.21	-6.61	-6.06	-6.17	-6.21
**Druglikeness**	Lipinski #violations	0	0	0	0	0	0	0
	Ghose #violations	0	0	0	0	0	0	0
	Veber #violations	0	0	0	0	0	0	0
	Egan #violations	0	0	0	0	0	0	0
	Muegge #violations	0	0	0	0	0	0	0
	Bioavailability Score	0.55	0.55	0.55	0.55	0.55	0.55	0.55

Table S1 (cont.) ADMET properties predictions by SwissModel.

Compound		Actinodaphine	Boldine	Coclaurine	Laurolitsine	Assimilobine	Reticuline	Bulbocapnine
**Medicinal Chemistry**	PAINS #alerts	0	0	0	0	0	0	0
	Brenk #alerts	0	0	0	0	0	0	0
	Leadlikeness #violations	0	0	0	0	0	0	0
	Synthetic Accessibility	3.56	3.65	2.78	3.46	3.24	3.07	3.77
**Toxicity**	Mutagenic	none	none	none	none	none	none	none
	Tumorigenic	none	none	high	none	none	none	none
	Reproductive Effective	none	none	none	none	none	none	none
	Irritant	none	none	none	none	none	none	none

Table S2: Score values and probability of activity of the test compounds on the target 3CL^{pro} (PDB: 6M2N).

Compound	Moldock Score	(*p*) Moldock Score	Rerank Score	(*p*) Rerank Score	PLANTS Score	(*p*) PLANTS Score	(*p*) Total
**actinodaphine**	-110.452	0.85	-87.5808	0.88	-495.574	0.88	0.87
**boldine**	-114.959	0.88	-91.2593	0.92	-497.582	0.88	0.89
**coclaurine**	-114.653	0.88	-92.7533	0.93	-479.268	0.85	0.89
**laurolitsine**	-115.519	0.88	-89.944	0.90	-488.491	0.87	0.89
**assimilobine**	-88.7997	0.68	-64.2278	0.64	-451.941	0.80	0.71
**reticuline**	**-129.865**	**1**	**-98.8927**	**1**	-460.433	0.82	**0.94**
**bulbocapnine**	-110.315	0.84	-83.281	0.84	-495.129	0.88	0.85
**Lig PDB/ positive Control**	-97.7095	0.75	-79.639	0.80	**-559.57**	**1**	0.85

Legend: In bold is the lowest score.

Table S3: Score values and probability of activity of the test compounds on the target 3CL^{pro} (PDB: 7B3E).

Compound	Moldock Score	(*p*) Moldock Score	Rerank Score	(*p*) Rerank Score	PLANTS Score	(*p*) PLANTS Score	(*p*) Total
**actinodaphine**	-84.0975	0.89	-30.5719	0.50	-419.077	0.79	0.72
**boldine**	-84.3368	0.89	**-60.9568**	**1**	-416.924	0.78	**0.89**
**coclaurine**	-78.2785	0.82	-34.228	0.56	-419.069	0.79	0.72
**laurolitsine**	-81.0543	0.85	-45.3966	0.74	-420.503	0.79	0.79
**assimilobine**	-70.4253	0.74	7.80961	-0.12	-402.672	0.76	0.45
**reticuline**	-93.9823	0.99	-50.4015	0.82	-426.781	0.80	0.87
**bulbocapnine**	**-94.3855**	**1**	-50.0182	0.82	-445.505	0.84	0.88
**Lig PDB/ positive Control**	-78.5492	0.83	1.72583	-0.02	**-528.57**	**1**	0.60

Legend: In bold is the lowest score.

Table S4: Score values and probability of activity of the test compounds on the target PL^{pro} (PDB: 7LBR).

Compound	Moldock Score	(*p*) Moldock Score	Rerank Score	(*p*) Rerank Score	PLANTS Score	(*p*) PLANTS Score	(*p*) Total
**actinodaphine**	-119.994	0.67	-95.1362	0.69	-300.432	0.50	0.62
**boldine**	-109.138	0.61	-72.0945	0.52	-295.071	0.49	0.54
**coclaurine**	-106.26	0.59	-78.2978	0.57	-315.275	0.53	0.56
**laurolitsine**	-112.807	0.63	-81.642	0.59	-289.608	0.48	0.57
**assimilobine**	-106.279	0.59	-78.6753	0.57	-284.505	0.47	0.54
**reticuline**	-118.242	0.66	-21.3136	0.15	-321.294	0.54	0.45
**bulbocapnine**	-123.147	0.68	-88.5518	0.64	-298.235	0.50	0.61
**Lig PDB/ positive Control**	**-178.71**	**1**	**-136.949**	**1**	**-592.812**	**1**	**1**

Legend: In bold is the lowest score.

Table S5: Score values and probability of activity of the test compounds on the target PL^{pro} (PDB: 7TZJ).

Compound	Moldock Score	(*p*) Moldock Score	Rerank Score	(*p*) Rerank Score	PLANTS Score	(*p*) PLANTS Score	(*p*) Total
**actinodaphine**	-103.473	0.83	-79.2322	0.77	-403.972	0.67	0.76
**boldine**	-96.5568	0.78	-61.6073	0.59	-371.097	0.62	0.66
**coclaurine**	-103.549	0.83	-79.0817	0.76	-401.086	0.67	0.76
**laurolitsine**	-97.008	0.78	-56.4308	0.54	-363.582	0.61	0.64
**assimilobine**	-84.8215	0.68	-67.0917	0.65	-370.901	0.62	0.65
**reticuline**	-123.467	1	-87.3236	0.84	-412.378	0.69	0.84
**bulbocapnine**	-119.546	0.96	-56.4875	0.54	-392.416	0.69	0.72
**Lig PDB/ positive Control**	**-123.096**	**0.99**	**-102.81**	**1**	**-594.722**	**1**	**0.99**

Legend: In bold is the lowest score.

Table S6: Score values and probability of activity of the test compounds on the target Spike (PDB: 3SCI).

Compound	Moldock Score	(*p*) Moldock Score	Rerank Score	(*p*) Rerank Score	PLANTS Score	(*p*) PLANTS Score	(*p*) Total
**actinodaphine**	-101.755	0.59	-59.4184	0.55	-62.6934	0.88	0.68
**boldine**	-108.076	0.63	-84.8481	0.79	-54.9883	0.77	0.73
**coclaurine**	-103.13	0.60	-80.0076	0.75	-62.9042	0.88	0.74
**laurolitsine**	-107.169	0.62	-84.9237	0.79	-53.1883	0.75	0.72
**assimilobine**	-86.2089	0.50	-69.1342	0.64	-55.0889	0.77	0.64
**reticuline**	-117.652	0.69	-79.3336	0.74	-58.0239	0.82	0.75
**bulbocapnine**	-112.808	0.66	-88.9631	0.83	-58.1751	0.82	0.77
**Lig PDB/ positive Control**	**-170.128**	**1**	**-106.666**	**1**	**-70.7053**	**1**	**1**

Legend: In bold is the lowest score.

Table S7: Score values and probability of activity of the test compounds on the target Spike (PDB: 6M0J).

Compound	Moldock Score	(*p*) Moldock Score	Rerank Score	(*p*) Rerank Score	PLANTS Score	(*p*) PLANTS Score	(*p*) Total
**actinodaphine**	-107.117	0.59	-85.5649	0.68	**-67.5166**	1	0.76
**boldine**	-101.282	0.56	-81.4506	0.65	-52.0767	0.77	0.66
**coclaurine**	-100.287	0.55	-68.1878	0.54	-65.7778	0.97	0.69
**laurolitsine**	-105.687	0.58	-80.1244	0.64	-58.3808	0.86	0.69
**assimilobine**	-85.3583	0.47	-64.8527	0.52	-61.7834	0.91	0.63
**reticuline**	-125.626	0.69	-95.6976	0.76	-60.9201	0.90	0.78
**bulbocapnine**	-107.579	0.59	-65.3193	0.52	-64.0248	0.94	0.69
**Lig PDB/ positive Control**	**-180.188**	**1**	**-124.414**	**1**	-61.688	**0.91**	**0.97**

Legend: In bold is the lowest score.
